# A cation diffusion facilitator, *GmCDF1*, negatively regulates salt tolerance in soybean

**DOI:** 10.1371/journal.pgen.1007798

**Published:** 2019-01-07

**Authors:** Wei Zhang, Xiliang Liao, Yanmei Cui, Weiyu Ma, Xinnan Zhang, Hongyang Du, Yujie Ma, Lihua Ning, Hui Wang, Fang Huang, Hui Yang, Guizhen Kan, Deyue Yu

**Affiliations:** 1 National Key Laboratory of Crop Genetics and Germplasm Enhancement, National Center for Soybean Improvement, Jiangsu Collaborative Innovation Center for Modern Crop Production, Nanjing Agricultural University, Nanjing, China; 2 Provincial Key Laboratory of Agrobiology, Institute of Agro-biotechnology, Jiangsu Academy of Agriculture Sciences, Nanjing, China; 3 School of Life Sciences, Guangzhou University, Guangzhou, China; Purdue University, UNITED STATES

## Abstract

Salt stress is one of the major abiotic factors that affect the metabolism, growth and development of plants, and soybean [*Glycine max* (L.) Merr.] germination is sensitive to salt stress. Thus, to ensure the successful establishment and productivity of soybeans in saline soil, the genetic mechanisms of salt tolerance at the soybean germination stage need to be explored. In this study, a population of 184 recombinant inbred lines (RILs) was utilized to map quantitative trait loci (QTLs) related to salt tolerance. A major QTL related to salt tolerance at the soybean germination stage named *qST-8* was closely linked with the marker Sat_162 and detected on chromosome 8. Interestingly, a genome-wide association study (GWAS) identified several single nucleotide polymorphisms (SNPs) significantly associated with salt tolerance in the same genetic region on chromosome 8. Resequencing, bioinformatics and gene expression analyses were implemented to identify the candidate gene *Glyma*.*08g102000*, which belongs to the cation diffusion facilitator (CDF) family and was named *GmCDF1*. Overexpression and RNA interference of *GmCDF1* in soybean hairy roots resulted in increased sensitivity and tolerance to salt stress, respectively. This report provides the first demonstration that *GmCDF1* negatively regulates salt tolerance by maintaining K^+^-Na^+^ homeostasis in soybean. In addition, *GmCDF1* affected the expression of two ion homeostasis-associated genes, salt overly sensitive 1 (*GmSOS1*) and Na^+^/H^+^ exchanger 1 (*GmNHX1*), in transgenic hairy roots. Moreover, a haplotype analysis detected ten haplotypes of *GmCDF1* in 31 soybean genotypes. A candidate-gene association analysis showed that two SNPs in *GmCDF1* were significantly associated with salt tolerance and that Hap1 was more sensitive to salt stress than Hap2. The results demonstrated that the expression level of *GmCDF1* was negatively correlated with salt tolerance in the 31 soybean accessions (*r* = -0.56, *P* < 0.01). Taken together, these results not only indicate that *GmCDF1* plays a negative role in soybean salt tolerance but also help elucidate the molecular mechanisms of salt tolerance and accelerate the breeding of salt-tolerant soybean.

## Author summary

Soil salinity can seriously threaten soybean growth and development and seed germination is a key phase in the soybean growth cycle. Thus, understanding the genetic mechanism of salt tolerance at the germination stage is very important for improving the salt tolerance of soybean at the germination stage. An analysis combining linkage mapping, GWAS and whole-genome sequencing of two soybean accessions revealed that the *GmCDF1* was associated with soybean salt tolerance. The transformation of soybean hairy roots and an inductively coupled plasma optical emission spectrometry (ICP-OES) analysis confirmed that *GmCDF1* negatively regulates salt tolerance by maintaining ion homeostasis in soybean. A haplotype analysis found ten haplotypes of *GmCDF1* in 31 soybean accessions, and a candidate gene association analysis identified two probable causative *GmCDF1* polymorphisms. Moreover, higher *GmCDF1* expression resulted in salt sensitivity. Our results not only reveal the role of *GmCDF1* in soybean adaptation to salt stress but also help elucidate the genetic molecular mechanisms of salt tolerance and facilitate the implementation of marker-assisted selection for salt tolerance in soybean breeding programs.

## Introduction

Soil salinity is a global ecological issue that has severely affected plant growth and development and decreased agricultural production. A high-salt environment causes various damages to crops, such as plant water loss, high osmotic stress, and homeostasis and ion imbalances in plant cells [1,2]. The cultivation of salinity-tolerant plants and improvements in their adaptability to saline-alkali soils are urgently needed. Understanding the mechanism of salt tolerance in plants is the most crucial basic knowledge needed for the breeding of salt-tolerant plants [3,4]. Soybean is a traditional edible leguminous crop that provides abundant protein, high-quality vegetable oils and a variety of physiologically active substances to human beings. In addition, soybean is a moderately salt-tolerant crop, and the yield of soybean is significantly reduced if the soil salinity exceeds 5 dS/m [5].

Plant salt tolerance is a complex quantitative trait that is affected by numerous genetic and nongenetic factors [6,7]. In recent years, both forward and reverse genetic approaches have been applied to reveal the functions of key salinity response genes in soybean. Biparental quantitative trait locus (QTL) mapping and genome-wide association studies (GWAS) have been used as effective and precise tools to detect QTLs associated with salt tolerance, and a number of QTLs for salt tolerance have been detected in previous studies [8–14]. For example, a major QTL was detected and mapped near the single sequence repeat (SSR) marker Sat_091 on chromosome 3, and this QTL explained 41% and 60% of the total phenotypic variation observed under greenhouse and field conditions, respectively, in an F_2:5_ population derived from a cross of the cultivars S-100 (salt-tolerant line) and Tokyo (salt-sensitive line) [11]. Other researchers recently confirmed this QTL in a different mapping population [8,9,13–15], and a gene at this major locus, *Glyma03g32900*, was identified and cloned [10,15–18].

Although the major QTLs related to salt tolerance on chromosome 3 were consistently mapped in several studies, some reports indicated that additional QTLs/genes contribute to salt tolerance in the soybean genome. For example, a previous study showed that 45 SNPs mapped on chromosomes 2, 3, 7, 8, 10, 13, 14, 16, and 20, including 31 SNPs on chromosome 3 mapped at or near the previously reported major salt tolerance QTL, are significantly associated with salt tolerance [19]. Through both field and greenhouse experiments, other researchers identified a major salt tolerance-related QTL on chromosome 18 in F_7:11_ recombinant inbred lines (RILs; 184) [20]. Our previous study found that eight SNPs and 13 suggestive SNPs are associated with salt tolerance indices and verified that five candidate genes located on chromosomes 8, 9 and 19 are associated with the response to salt stress at the soybean germination stage by association mapping [21]. A novel QTL associated with the leaf sodium content (LSC), which showed a high logarithm of odds (LOD) value (4.56) and *R*^2^ (11.5%), was identified on chromosome 13 using the soybean physical map of 132 F_2_ plants [13].

The above studies largely focused on salt tolerance at the soybean seedling stage but rarely investigated salt tolerance at the germination stage. However, seed germination is critical for ensuring new seedlings and enhancing yield during the plant growth cycle. It has been suggested that soybean germplasms have different degrees and mechanisms of salt tolerance at different developmental stages [5]. Although QTLs for salt stress at the germination stage have been identified by linkage mapping [22,23] and association analysis [21,24], there is little information regarding the genetic mechanisms of salt tolerance at the germination stage. Understanding the genetic mechanism of salt tolerance at the seed germination stage is very important for improving the salt tolerance of soybean. In this study, linkage mapping and genome-wide association study were performed to dissect the genetic architecture of salt tolerance at the soybean seed germination stage, and a major QTL, *qST-8*, was found to be significantly associated with salt tolerance. Furthermore, an investigation combining whole-genome sequence, bioinformatics, and gene expression analyses as well as plant transformation demonstrated that a cation diffusion facilitator (CDF), *GmCDF1*, negatively regulates salt tolerance through Na^+^-K^+^ homeostasis in soybean. Additionally, haplotype and candidate-gene association analyses in 31 natural soybean varieties confirmed that *GmCDF1* plays a negative regulatory role in salt tolerance.

## Results

### Phenotypic variation and correlation analysis

The means, standard deviations and ranges of four germination-related traits—imbibition rate (IR), germination index (GI), germination potential (GP) and germination rate (GR)—and four salt tolerance indices—the ratio of the imbibition rate under salt conditions to the imbibition rate under no-salt conditions (ST-IR), the ratio of the germination index under salt conditions to the germination index under no-salt conditions (ST-GI), the ratio of the germination potential under salt conditions to the germination potential under no-salt conditions (ST-GP) and the ratio of the germination rate under salt conditions to the germination rate under no-salt conditions (ST-GR) of RILs and natural populations—are presented (S1 Table). The mean values of the four germination-related traits obtained in the presence of 150 mM NaCl were lower than found under normal conditions, which indicated that salt stress depressed the growth and development of soybean at the germination stage. Moreover, the level of salt tolerance-related traits, with the exception of ST-IR, varied widely in both the RILs and natural populations. The mean ST-IR, ST-GI, ST-GP and ST-GR varied from 0.85 to 0.99, from 0.25 to 1.02, from 0.20 to 0.88 and from 0.41 to 1.00, respectively, in the RILs population and from 0.86 to 0.97, from 0.21 to 0.88, from 0.00 to 0.92 and from 0.24 to 1.09, respectively, in the natural population. Two parents of the RILs population, Kefeng No.1 and Nannong 1138–2, showed different tolerances to salt stress (S1 Table). The ST-GR and ST-GI of Kefeng No.1 were higher than those of Nannong 1138–2, indicating that Nannong 1138–2 was more sensitive to salt stress than Kefeng No.1 at the germination stage. An analysis of variance (ANOVA) showed that the genotype, environment and the genotype-by-environment interaction significantly influenced the four salt tolerance indices (*P<*0.001) in the two populations (S1 Table). In addition, significant (*P*<0.001) genetic variations were found for the four germination-related traits and the salt tolerance indices in the two populations across four and three environments, respectively. Moreover, the phenotypic frequencies of ST-IR, ST-GI, ST-GP and ST-GR in the two populations approximately fit a normal continuous distribution, indicating that these four salt tolerance indices are quantitative traits controlled by multiple factors (S1 Fig).

Pearson’s correlations among the four salt tolerance indices were analyzed based on the means of two populations (S2 Table). For the two populations, ST-IR was significantly negatively correlated with ST-G, ST-GP and ST-GR (*P*<0.01), whereas ST-GI was significantly positively correlated with ST-GP and ST-GR, and ST-GP was strongly positively correlated with ST-GR (*P*<0.01).

### QTLs for salt tolerance at the germination stage

**Fig 1:**
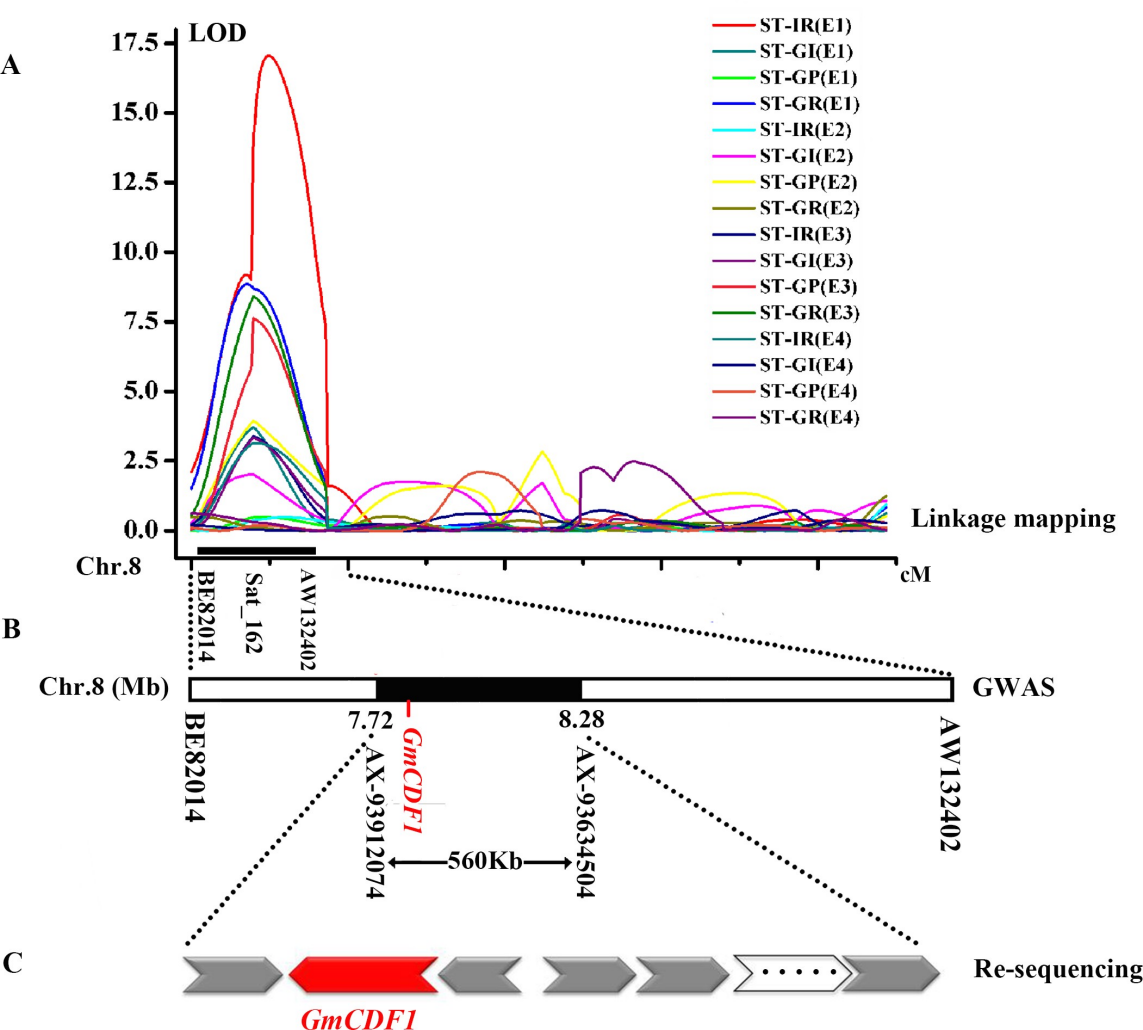
Identification of *GmCDF1* through an analysis combining linkage mapping, GWAS and resequencing. **(A)** *qST-8* (QTL related to salt tolerance on chromosome 8) was identified in the samples of 184 recombinant inbred lines (RILs) collected from all four years and mapped to the interval between the markers BE82014 and AW132402 by linkage mapping. **(B)** This QTL was further narrowed down to a physical region of 560 kb between the SNPs AX-94047897 and AX-93634504 on chromosome 8 by GWAS. **(C)** Resequencing was performed to detect the different genes located within the region identified in **(B)** between the two parents of the RILs population, Kefeng No. 1 and Nannong 1138–2.

Four salt tolerance indices of the RILs population in four different environments were used for QTL mapping. A total of 25 QTLs associated with four salt tolerance indices during the soybean germination stage were detected on chromosomes 1, 2, 7, 8, 10, 15, 17 and 18, with LOD values ranging from 2.50 to 17.06 (Table 1). Three of the five QTLs for ST-IR, two of the four QTLs for ST-GI, three of the nine QTLs for ST-GP and two of the seven QTLs for ST-GR were significantly associated with salt tolerance and located on chromosome 8. With the exception of *qSTGP-8-2*, other QTLs related to salt tolerance and located on chromosome 8 (named *qST-8*) that showed largely overlapping confidence intervals (CIs) were considered the same QTL. This QTL closely linked with the marker Sat_162 was detected mostly for the four salt tolerance indices in E1, E2, E3, and E4, contained the physical genetic region between the markers BE820148 and AW132402 (Fig 1A) and explained 6.25%–46.82% of the phenotypic variation. The marker BE820148 was detected for ST-GR once in E1 with significant LOD (8.85) and R^2^ (12.84%) values close to the marker Sat_162. This result suggested that the candidate gene might be located within the region between markers BE820148 and Sat_162 or closer to marker Sat_162.

**Table 1:** QTLs for salt tolerance-related traits based on the mean traits of three replications in 184 recombinant inbred lines.

**Trait**	**Chr.**	**Env.**	**LOD**	**Marker**	**R**^**2**^**(%)**	**Pos.(cM)**	**QTLs**	**MI**
ST-IR	2	E4	3.23	satt266	7.03	168.9	*qSTIR-2*	satt428-Rsc_7
	7	E3	3.01	satt245	10.69	53.4	*qSTIR-8*	satt590-sat_148
	8	E1	17.06	**sat_162**	46.82	24.8	*qSTIR-8*	BE820148-AW132402
	8	E3	3.39	**sat_162**	6.94	19.8	*qSTIR-8*	BE820148-AW132402
	8	E4	3.14	**sat_162**	6.73	19.8	*qSTIR-8*	BE820148-AW132402
ST-GI	8	E1	3.72	**sat_162**	8.46	19.8	*qSTGI-8*	BE820148-AW132402
	8	E3	3.34	**sat_162**	7.11	19.8	*qSTGI-8*	BE820148-AW132402
	15	E3	2.64	satt606	6.02	42.7	*qSTGI-15*	satt606-sat_331
	17	E1	2.56	satt669	10.94	77.7	*qSTGI-17*	satt669-sat_292
ST-GP	1	E3	4.12	satt468	22.95	49.3	*qSTGP-1-2*	satt468-sat_160
	2	E1	3.21	satt611	9.23	114.2	*qSTGP-2*	satt611-satt428
	7	E3	3.04	satt245	9.08	57.4	*qSTGP-7*	satt590-sat_148
	8	E2	3.94	**sat_162**	8.66	19.8	*qSTGP-8-1*	BE820148-AW132402
	8	E3	7.63	**sat_162**	15.61	19.8	*qSTGP-8-1*	BE820148-AW132402
	8	E2	2.84	sat_310	6.25	112	*qSTGP-8-2*	sat_310-sat_232
	10	E1	4.44	satt331	33.4	55.9	*qSTGP-10*	satt331-sat_196
	17	E1	3.04	satt669	16.98	79.7	*qSTGP-17-1*	satt669-sat_292
	17	E2	2.85	sat_222	6.08	99.5	*qSTGP-17-2*	satt669-sat_292
ST-GR	1	E3	3.7	satt468	13.12	62.3	*qSTGR-1*	satt468-sat_160
	7	E3	3.15	satt590	16.55	36.2	*qSTGR-7*	satt590-sat_148
	8	E1	8.85	BE820148	22.41	18	*qSTGR-8*	BE820148-AW132402
	8	E3	8.41	**sat_162**	16.66	19.8	*qSTGR-8*	BE820148-AW132402
	10	E4	4.8	sat_274	10.29	30.6	*qSTGR-10*	sat_231-satt331
	17	E1	3.93	att669	13.73	80	*qSTGR-17*	satt669-sat_292
	18	E1	2.5	sat_358	6.74	47.6	*qSTGR-18*	sat_358-sat_290

Chr: chromosome; CI: confidence interval; Env: environment; MI: marker interval; Pos: position; R^2^: percentage of phenotypic variation explained by the QTL; ST: salt tolerance; ST-IR: ratio of the imbibition rate under salt conditions to the imbibition rate under no-salt conditions; ST-GI: ratio of the germination index under salt conditions to the germination index under no-salt conditions; ST-GP: ratio of the germination potential under salt conditions to the germination potential under no-salt conditions; and ST-GR: ratio of the germination rate under salt conditions to the germination rate under no-salt conditions.

### GWAS for salt tolerance-related traits in a natural population

**Fig 2:**
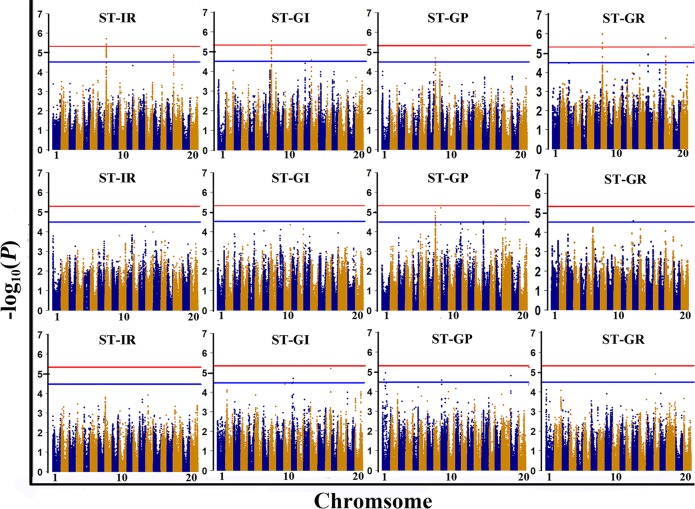
Manhattan plots for the GWAS results of the ST-IR, ST-GI, ST-GP, and ST-GR in three environments (E1, E2, and E3). The three rows from the top to the bottom show the Manhattan plots for the GWAS results of the four salt tolerance indices in E1, E2 and E3, respectively. The red line indicates the significance threshold (-log_10_(*P*) = 5.32), and the blue line indicates the suggestive threshold (-log_10_(*P*) = 4.5). The abscissa axis presents the chromosomes of soybean from 1 to 20 in blue or orange.

A GWAS was conducted to detect SNPs associated with salt tolerance across three environments with 207,608 SNPs [minor allele frequency (MAF)>0.05] obtained from the NJAU 355K SoySNP array [25], and the Manhattan and quantile-quantile plots for the GWAS results are shown in Fig 2 and S2 Fig. The 18 SNPs significantly (with a significance threshold of -log_10_(*P*)≥5.32) associated with salt tolerance indices are listed in Table 2. In addition, 74 SNPs with suggestive thresholds (4.5≤-log_10_(*P*)<5.32) were also identified in the GWAS (S3 Table). These SNPs are located on chromosomes 1, 8, 11, 13, 14, 15, 16, 18 and 19, indicating that the salt tolerance of soybean at the germination stage is controlled by multiple genes. Moreover, we found that 17 out of 18 significant SNPs and 48 out of 74 suggestive SNPs were located on chromosome 8, forming a cluster flanked by the SNP markers AX-93912074 and AX-93634504 (-log_10_(*P*)≥5.32) with a physical position of 7716458–8268861 bp. Interestingly, this cluster was located in *qST-8*, which was identified by the previous linkage mapping in four environments (Fig 1B), indicating that this cluster is critical for salt tolerance at the germination stage of soybean and that a candidate gene for salt tolerance can likely be identified.

**Table 2:** Details of SNPs significantly associated with salt tolerance indices of soybean (with a significance threshold of -log_10_(*P*)≥5.32).

Traits	SNP	Chromosome	Position	-log_10_(*P*)	R^2^(%)
**ST-IR**	AX-93751763	8	8078697	5.40	15.15
	AX-94048075	8	8166922	5.40	15.17
	AX-94048076	8	8184086	5.32	14.94
	AX-94048081	8	8193755	5.32	14.94
	AX-94048086	8	8204675	5.72	16.02
	AX-94048102	8	8236728	5.32	14.94
	AX-93634502	8	8240007	5.32	14.94
	AX-93751824	8	8241205	5.44	15.28
	AX-94048104	8	8247379	5.40	15.17
	AX-94048105	8	8251019	5.40	15.17
	AX-93751829	8	8255498	5.32	14.94
	AX-93751830	8	8258464	5.44	15.28
	AX-93751831	8	8260590	5.32	14.94
	AX-93634504	8	8268861	5.39	15.13
**ST-GI**	AX-93751824	8	8241205	5.54	19.10
**ST-GR**	AX-93912074	8	7716458	5.99	25.70
	AX-94047897	8	7743527	5.51	24.48
	AX-93656763	18	6117413	5.78	25.15

R^2^: percentage of phenotypic variation explained by the SNP;ST: salt tolerance; ST-IR: ratio of the imbibition rate under salt conditions to the imbibition rate under no-salt conditions; ST-GI: ratio of the germination index under salt conditions to the germination index under no-salt conditions; ST-GP: ratio of the germination potential under salt conditions to the germination potential under no-salt conditions; and ST-GR: ratio of the germination rate under salt conditions to the germination rate under no-salt conditions.

### A candidate gene for salt tolerance was identified by whole-genome sequencing and expression analysis

According to gene annotation on Phytozome 12 (https://phytozome.jgi.doe.gov/pz/portal.html), 70 gene models are located within the above-described cluster. For fine mapping, we performed whole-genome sequencing on Kefeng No.1 and Nannong 1138–2, which are the parents of the RILs population used in this study, and the SNP density distribution within the soybean genome is shown in S2 Fig. We compared the whole genome of Kefeng No.1 to that of Nannong 1138–2 and found that 273 SNPs were located on chromosome 8 between the SNP markers AX-93912074 and AX-93634504. Among these SNPs, 42 nonsynonymous SNPs were located in the exons of 21 genes, and 15 SNPs were located in the 2.0-kb promoter regions of 11 genes, including three identical genes (S4 Table). We performed a quantitative real-time PCR (qRT-PCR) analysis to investigate whether the expression of these 29 genes (S4 Table) in Kefeng No.1 and Nannong 1138–2 was affected by salt stress. The results demonstrated that the expression of six genes (*Glyma*.*08g101300*, *102200*, *103000*, *106100*, *106200* and *106400*) was too low to be detected, whereas that of 16 genes did not change significantly in response to salt stress (S3 Fig), and that of seven genes could be induced by salt stress (S4 Fig) at the soybean germination stage. Among these seven genes, six were induced by salt stress in both Kefeng No.1 and Nannong 1138–2, and only *Glyma*.*08g102000* was dramatically upregulated in Nannong 1138–2 but not in Kefeng No.1 under salt stress. In fact, the expression level of *Glyma*.*08g102000* in Nannong 1138–2 was nearly 30-fold higher than that in Kefeng No.1 after treatment with 150 mM NaCl, whereas only a 1-3-fold change was found for the other six genes. Thus, *Glyma*.*08g102000*, which is located within the QTL *qST-8* detected in our study, might be a candidate gene involved in the regulation of salt tolerance in soybean.

### *Glyma*.*08g102000* encodes a CDF and shows differential expression in tolerant and susceptible soybeans under salt stress

*Glyma*.*08g102000* encodes an 817-amino-acid protein, has a length of 2457 bp and is a member of the CDF family (named *GmCDF1*). A phylogenetic analysis indicated a close relationship between *Glyma*.*08g102000* and *AtMTP12* (S6A Fig). The TMHMM program (http://www.cbs.dtu.dk/services/TMHMM/) predicted that *GmCDF1* possesses 14 transmembrane domains (TMs) with cytosolic N and C termini, similarly to *AtMTP12* (S6B Fig). A previous report showed that *AtMTP12* forms a functional complex with *AtMTP5t1* to transport Zn into the Golgi and thereby increases tolerance to Zn stress in *Arabidopsis* [26]. However, another study found that in rice, *Os08g32650* and *Os01g03914*, two homologous genes of *GmCDF1*, are responsive to salt stress, and the expression levels of these two genes are lower in two salt-tolerant mutant lines of rice than in sensitive wild-type plants under salt stress [27]. These results suggest that *GmCDF1* might encode a cation diffusion facilitator and respond to salt stress.

**Fig 3:**
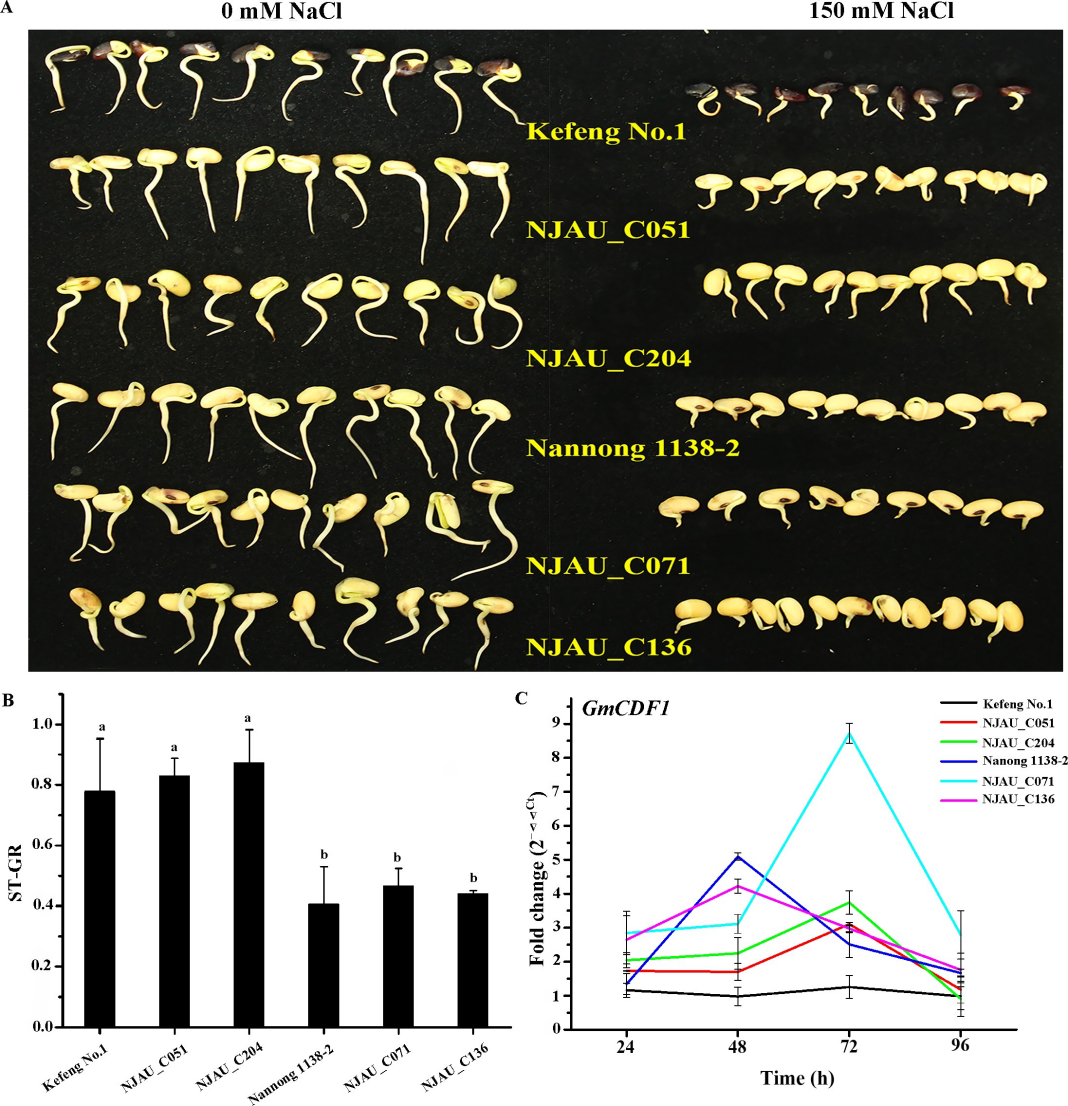
Germination of six soybean cultivars and expression of *GmCDF1* under normal or salt stress conditions. (A) The salt-tolerant soybean cultivars (Kefeng No.1, NJAU_C051 and NJAU_C204) performed better than the salt-sensitive cultivars (Nannong 1138–2, NJAU_C071 and NJAU_C136) under salt stress. (B) The ST-GR values of the salt-sensitive soybean cultivars (Nannong 1138–2, NJAU_C071 and NJAU_C136) were lower than those of the salt-tolerant cultivars (Kefeng No.1, NJAU_C051 and NJAU_C204). Different letters at the top of each column indicate significant differences, as determined by ANOVA (p < 0.05). The data are presented as the means±SEs. (C) Expression analysis of *GmCDF1* in soybean cultivars (Kefeng No. 1, NJAU_C051, NJAU_C204, Nannong 1138–2, NJAU_C071 and NJAU_C136) after treatment with 150 mM NaCl for 24 h, 48 h, 72 h and 96 h at the germination stage. The data are presented as the means±SEs.

We performed a qRT-PCR analysis to explore the expression pattern of *GmCDF1* in six representative soybean accessions, including three salt-tolerant accessions (Kefeng No.1, NJAU_C051 and NJAU_C204) and three salt-sensitive accessions (Nannong 1138–2, NJAU_C071 and NJAU_C136), at the germination stage under normal and salt stress conditions (Fig 3A and 3B). As shown in Fig 3C, the expression of *GmCDF1* in Kefeng No.1 was not significantly enhanced after exposure to salt stress. In contrast, the expression of *GmCDF1* in NJAU_C051 and NJAU_C204, the other two salt-tolerant soybean accessions, was upregulated from 48 h and reached a peak value at 72 h. In Nannong 1138–2 and NJAU_C136, the expression of *GmCDF1* was upregulated after NaCl treatment for 24 h and reached a peak value at 48 h, and in NJAU_C071, *GmCDF1* was upregulated after NaCl treatment for 24 h and reached a peak value at 72 h. Interestingly, higher fold-changes in the expression of *GmCDF1* after treatment with salt for 48 and 96 h were observed in the salt-sensitive accessions than in the salt-tolerant accessions (Fig 3C).

To further confirm the candidate gene, we investigated the expression patterns of *GmCDF1* in different soybean tissues, and our results showed that *GmCDF1* was expressed constitutively in most soybean tissues. The highest level of *GmCDF1* transcript was detected in flowers, seeds and roots, whereas *GmCDF1* was weakly expressed in leaves, pods and stems (S7 Fig). The high expression level found in roots suggests that the function of *GmCDF1* could be investigated using the soybean hairy root transformation system [28].

### Overexpression of *GmCDF1* depresses salt tolerance in soybeans

To investigate the role of *GmCDF1* under salt stress, two constructs (pMDC83-*GmCDF1* and pBI-*GmCDF1*) were generated for overexpression (*GmCDF1*-OE) and RNA interference (*GmCDF1*-RNAi) analyses, respectively. Transgenic soybean hairy roots were produced using to the *Agrobacterium rhizogenes*-mediated hairy root transformation system [28]. The average expression level of *GmCDF1* in *GmCDF1*-OE hairy roots was 31.8-fold higher than that in the wild-type strain K599-generated (harboring the empty vector pMDC83) control hairy roots (Control 1), whereas the expression level of *GmCDF1* in the *GmCDF1*-RNAi hairy roots was 53% lower compared with that in the control hairy roots (Control 2) generated by strain K599 [harboring the empty vector pB7GWIWG2(II)] (S8A and S8B Fig).

**Fig 4:**
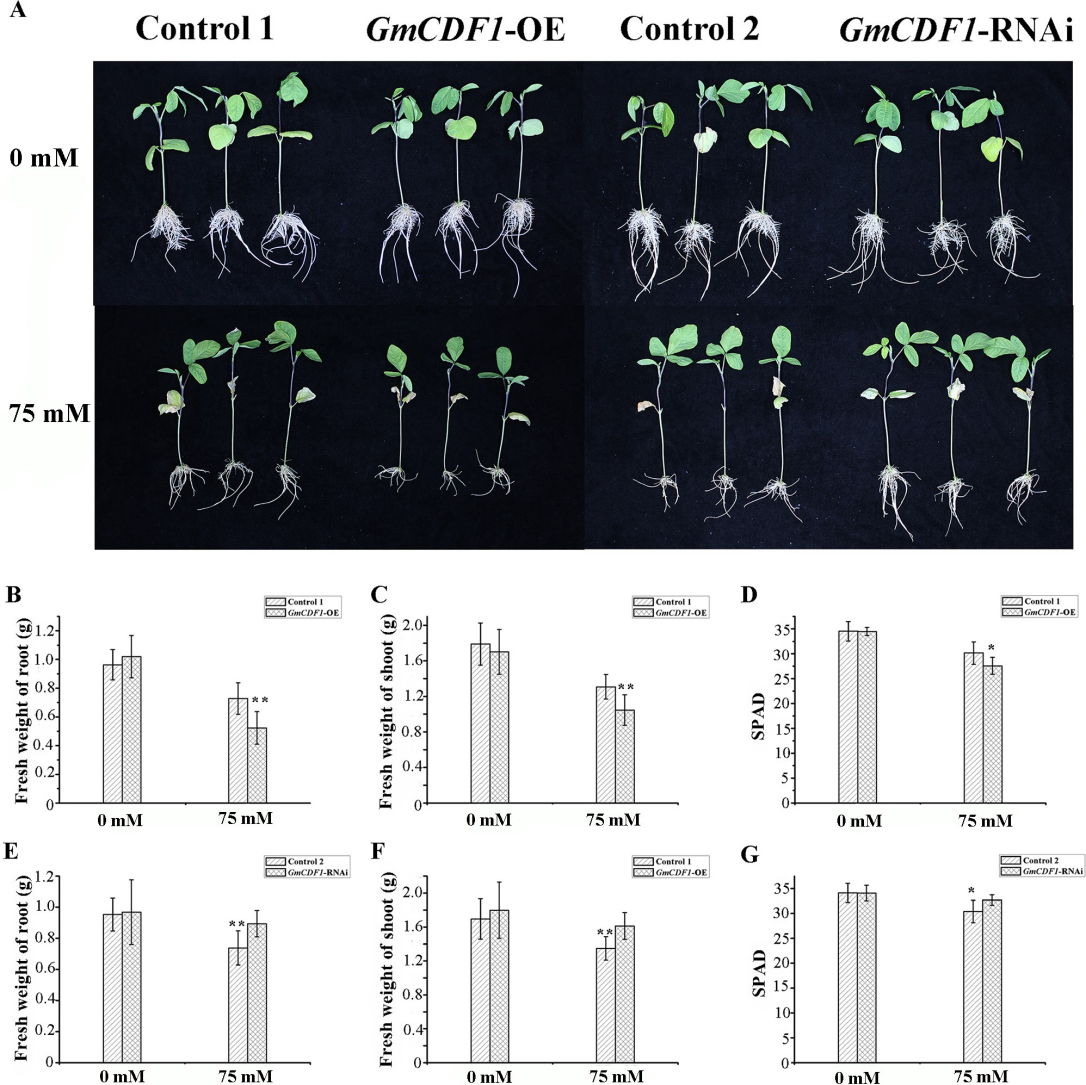
Performance of *GmCDF1* transgenic hairy roots under salt stress. **(A)** Growth of transgenic hairy roots and non-transgenic shoots treated with 0 mM or 75 mM NaCl for four days. More than 60 samples were analyzed, and three typical lines were selected. *GmCDF1*-OE: soybean hairy roots overexpressing *GmCDF1* Control 1: soybean hairy roots with the empty vector pMDC83; *GmCDF1*-RNAi: soybean hairy roots in which *GmCDF1* is silenced; Control 2: soybean hairy roots with the empty vector pB7GWIWG2(II). **(B)** Fresh weight of *GmCDF1*-OE and Control 1 hairy roots, **(C)** fresh weight of *GmCDF1*-OE and Control 1 shoots, and **(D)** SPAD values of their non-transgenic leaves treatment with 0 mM or 75 mM NaCl for four days. The data are presented as the means±SEs (n≥5). **(E)** Fresh weight of *GmCDF1*-RNAi and Control 2 hairy roots, **(F)** fresh weight of *GmCDF1*-RNAi and Control 2 shoots and **(G)** SPAD values of their non-transgenic leaves treatedwith 0 mM or 75 mM NaCl for four days. The data are presented as the means±SEs (n≥5). Statistical significance was detected by a two-tailed t-test. (**P<0.01, ***P<0.001).

In the presence of 0 mM NaCl, no apparent difference was found between the transgenic hairy roots and their controls, indicating that the overexpression or silencing of *GmCDF1* had little impact on the growth of soybean hairy roots under normal conditions (Fig 4A). However, after exposure to 75 mM NaCl for four days, obvious differences were observed between the transgenic plants and their controls. The *GmCDF1*-OE plants exhibited more sensitivity to salt stress than the Control 1 plants (Fig 4A). The average fresh weight of the Control 1 hairy roots and shoots was significantly heavier than that of the *GmCDF1*-OE hairy roots (Fig 4B and 4C). The *GmCDF1*-OE plants exhibited unhealthier leaves with lower average chlorophyll contents (soil plant analysis development, SPAD) than the Control 1 plants (Fig 4D). Moreover, the expression of *GmCDF1* was higher in the *GmCDF1*-OE hairy roots than in the Control 1 roots after treatment with NaCl for four days (S8A Fig).

Both the fresh weight of the hairy roots and shoots and the average SPAD value of the leaves of the *GmCDF1*-RNAi plants were higher than those of the Control 2 plants after treatment with 75 mM NaCl for four days (Fig 4E–4G). Under salt stress, the expression of *GmCDF1* was substantially suppressed in the *GmCDF1*-RNAi hairy roots compared with that in the Control 2 plants (S8B Fig). These overexpression and silencing experimental results suggest that *GmCDF1* negatively regulates salt tolerance in soybean.

### *GmCDF1* participates in maintaining ion homeostasis of Na^+^ and K^+^

**Fig 5:**
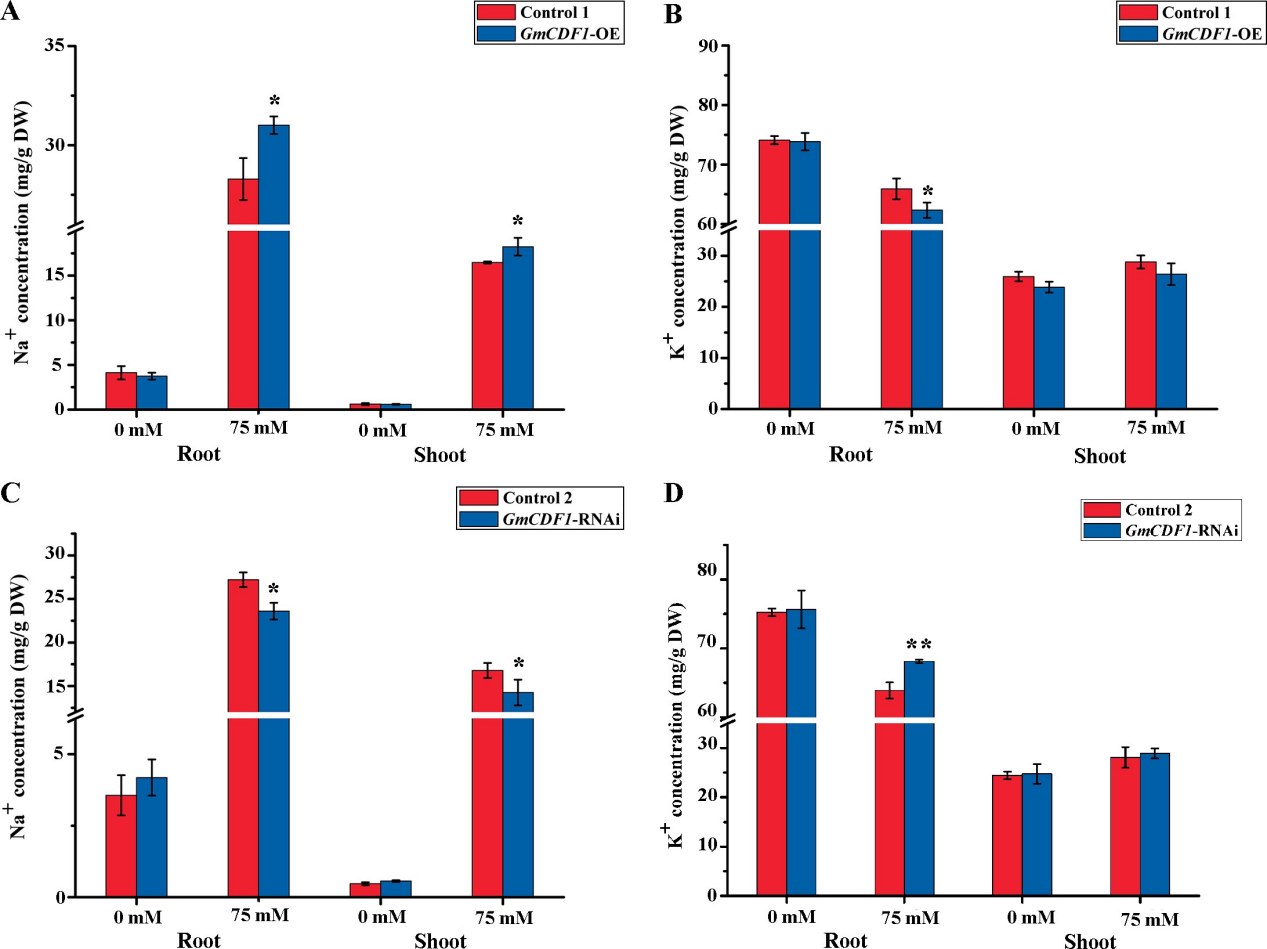
Distribution of Na^+^ and K^+^ in soybean hairy roots and shoots under salt stress. **(A)** Na^+^ contents of *GmCDF1*-OE hairy roots and their non–transgenic shoots compared with those of Control 1 plants after treatment with 0 mM or 75 mM NaCl for four days. The data are presented as the means±SEs (n≥5). **(B)** K^+^ contents of *GmCDF*1-OE hairy roots and their non–transgenic shoots compared with those of Control 1 plants after treatment with 0 mM or 75 mM NaCl for four days. The data are presented as the means±SEs (n≥5). **(C)** Na^+^ contents of *GmCDF1*-RNAi hairy roots and their non–transgenic shoots compared with those of Control 2 plants after treatment with 0 mM or 75 mM NaCl for four days. The data are presented as the means±SEs (n≥5). **(D)** K^+^ contents of *GmCDF1*-RNAi hairy roots and their non–transgenic shoots compared with those of Control 2 plants after treatment with 0 mM or 75 mM NaCl for four days. The data are presented as the means±SEs (n≥5). Statistical significance was detected by a two-tailed t-test. (*P<0.05, **P<0.01).

Excessive accumulation of salt usually leads to ion toxicity, which disrupts the metabolism of plants under salt stress. To assess the potential differences in the ion contents between the transgenic hairy roots and their corresponding control hairy roots, the ion contents of Na^+^ and K^+^ were determined by ICP-OES in this study. The results showed that in the absence of salt stress, the transgenic hairy roots showed little variation in the average Na^+^ and K^+^ contents compared with the corresponding control hairy roots, and the non-transgenic shoots exhibited similar results (Fig 5A–5D). However, after treatment with 75 mM NaCl for four days, the average Na^+^ contents in the *GmCDF1*-OE hairy roots and non-transgenic shoots were significantly higher than those found in the Control 1 hairy roots and shoots (Fig 5A), whereas the *GmCDF1*-RNAi hairy roots and non-transgenic shoots accumulated less Na^+^ than the Control 2 hairy roots and shoots (Fig 5C). Moreover, the *GmCDF1*-RNAi hairy roots accumulated less K^+^ than the Control 1 roots under salt stress (Fig 5B), whereas the *GmCDF1*-RNAi hairy roots accumulated more K^+^ than the Control 2 roots (Fig 5D).

### Expression of two salt tolerance-related genes is affected significantly by *GmCDF1* in transgenic hairy roots

**Fig 6:**
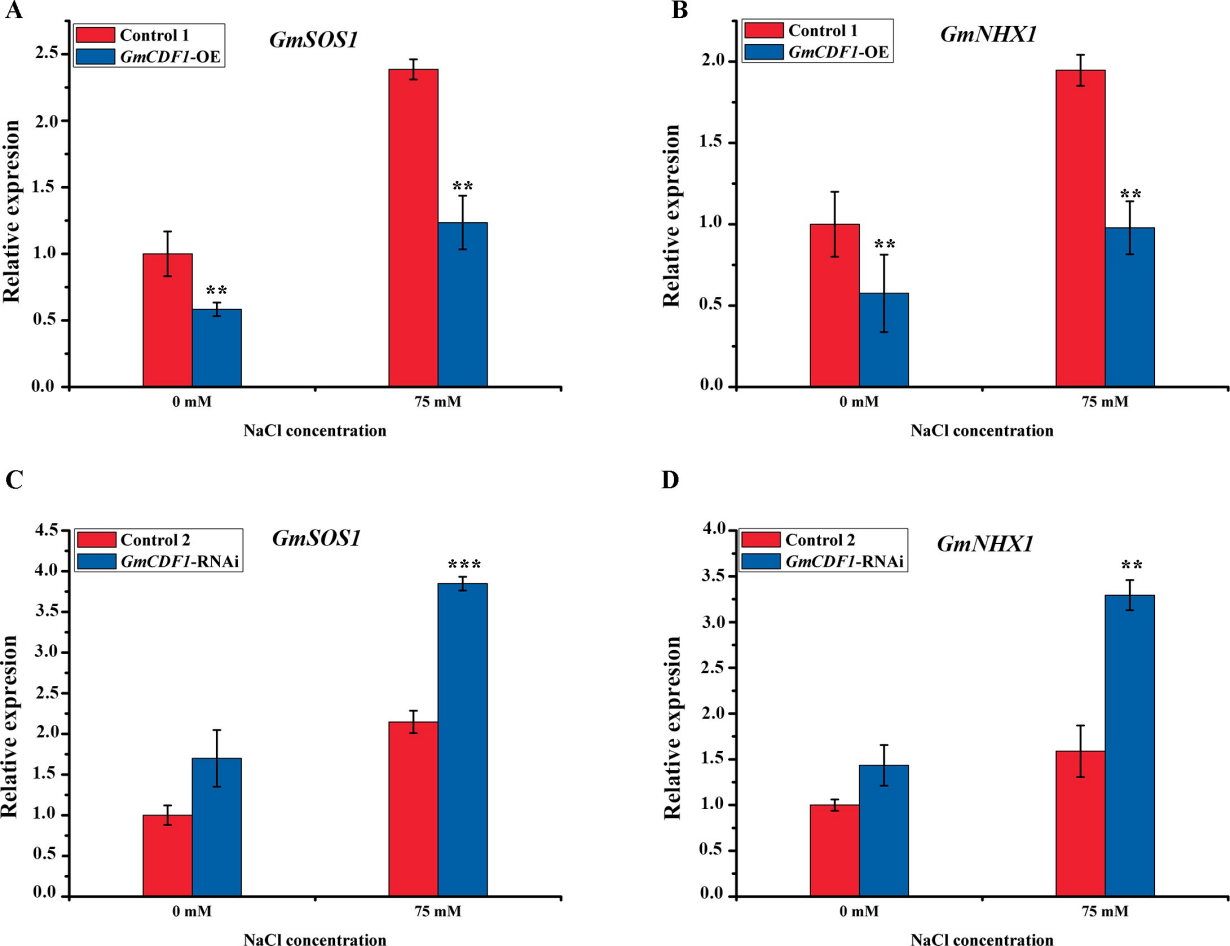
Relative expression levels of *GmSOS1* and *GmNHX1* in soybean hairy roots. (**A**) Relative expression levels of *GmSOS1* in Control 1 and *GmCDF1*-OE hairy roots after treatment with normal or salt conditions for four days. The data are presented as the means±SEs (n≥3). Statistical significance was detected by a two-tailed t-test. (**B**) Relative expression levels of *GmNHX1* in Control 1 and *GmCDF1*-OE hairy roots after treatment with normal or salt conditions for four days. The values are the means±SEs (n≥3). Statistical significance was detected by a two-tailed t-test. (**C**) Relative expression levels of *GmSOS1* in Control 2 and *GmCDF1*-RNAi hairy roots after treatment with normal or salt conditions for four days. The data are presented as the means±SEs (n≥3). Statistical significance was detected by a two-tailed t-test. (**D**) Relative expression levels of *GmNHX1* in Control 2 and *GmCDF1*-RNAi hairy roots after treatment with normal or salt conditions for four days. The data are presented as the means±SEs (n≥3). Statistical significance was detected by a two-tailed t-test. (*P<0.05, **P<0.01, ***P<0.001).

To further investigate the role of *GmCDF1* in salt stress adaptation in soybean, we analyzed the expression levels of salt stress-related genes with or without NaCl treatment. The relative expression of *GmSOS1* in the *GmCDF1*-OE hairy roots was significantly lower than that in the Control 1 hairy roots, under both normal and salt stress conditions. Similar to *GmSOS1*, the expression of *GmNHX1* in the *GmCDF1*-OE hairy roots was significantly lower than that in the Control 1 hairy roots under both normal and salt stress conditions (Fig 6B). In contrast, higher transcript levels of *GmSOS1* and *GmNHX1* were found in the *GmCDF1*-RNAi hairy roots compared with their control hairy roots, regardless of the presence of salt stress (Fig 6C and 6D). These results suggest that the overexpression or silencing of *GmCDF1* might affect the expression of *GmSOS1* and *GmNHX1* in soybean.

### Polymorphisms of *GmCDF1* are associated with salt tolerance in soybean

Because the expression level of *GmCDF1* in Nannong 1138–2 was nearly 30-fold higher than that in Kefeng No.1 after treatment with 150 mM NaCl for 48 h, differences might exist between the promoter regions of *GmCDF1* in Kefeng No.1 and Nannong 1138–2. Thus, the 2.0-kb promoter regions of *GmCDF1* upstream of the start codon were cloned and sequenced, and the results showed that the promoter of *GmCDF1* in Kefeng No.1 was 747 bp shorter than that in Nannong 1138–2. In fact, seven deletions were identified in the promoter region of *GmCDF1* in Kefeng No.1, and these were located at -130 bp, -315~-319 bp, -736~-962 bp, -968~-985 bp, -994~-1318 bp, -1311~-1463 bp and -1469~-1686 bp (upstream of the start codon). These deletions in the promoter regions of *GmCDF1* might be the reason for the dramatic upregulation of *GmCDF1* in Nannong 1138–2 but not in Kefeng No.1 under salt stress.

**Fig 7:**
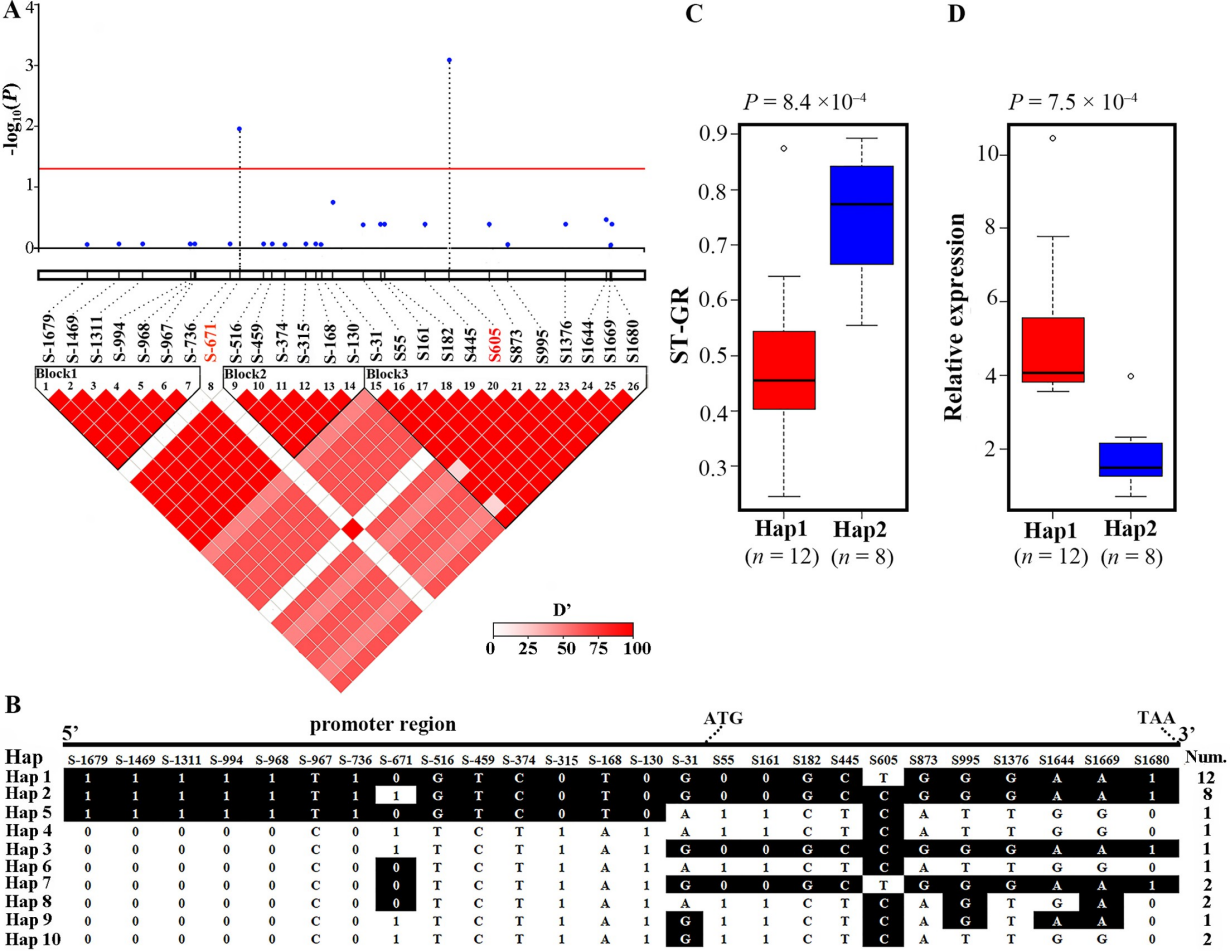
Natural variations in *GmCDF1* are significantly associated with salt tolerance indices in 31 soybean accessions. **(A)** *GmCDF1*-based association mapping and pairwise LD analysis (bottom) for *GmCDF1*. The red solid line represents the significance threshold (−log_10_(*P*) = 1.32). The blue dots above the red solid line represent significant variants for ST-GR (*P* < 0.05), and these are connected to the pairwise LD diagram with a black dashed horizontal line. The inverted triangle is the linkage disequilibrium (LD) plot between the *GmCDF1* SNPs. The physical position of each SNP is shown above the plot. The magnitude of LD indexed by the D’ statistic is shown. **(B)** Haplotypes of *GmCDF1* in a subset of 31 soybean accessions. An approximately 4.6-kb genomic region, including the 2.0-kb promoter region of *GmCDF1* upstream of the start codon and the 2.6-kb region of GmCDF1 from the 5'-UTR to the 3'-UTR, was used for the haplotype analysis. The haplotypes are displayed as a linear combination of alleles, and ten haplotypes were identified. The major alleles on each polymorphic site are highlighted in black. **(C)** Comparison of the ST-GR between haplotypes Hap1 and Hap2. *n* denotes the genotype number of the two haplotypes. Statistical significance was detected by a two-tailed t-test. **(D)** Comparison of the relative expression of *GmCDF1* between haplotypes Hap1 and Hap2. *n* denotes the genotype number of the two haplotypes. Statistical significance was detected by a two-tailed t-test.

We also sequenced the *GmCDF1* gene, an approximately 4.6-kb genomic region including the 2.0-kb promoter region of *GmCDF1* upstream of the start codon and the 2.6-kb region of *GmCDF1* from the 5'-UTR to 3'-UTR, in a subset of 31 soybean accessions representing varieties with high salt tolerance, moderate salt tolerance and low salt tolerance. The sequencing analysis identified 11 indels and 15 SNPs (MAF>0.05) (S5 Table) that were retained for the subsequent association analysis. After sequencing, five of the 15 SNPs and three of the 11 indels were identified as nonsynonymous mutations, and the remaining 10 SNPs and eight indels were found to be synonymous mutations (S5 Table). Furthermore, these 11 indels and 15 SNPs exhibited strong linkage disequilibrium (LD) and could form three LD blocks, as demonstrated by a LD analysis (Fig 7A). Furthermore, a *GmCDF1*-based association analysis was performed to investigate the relationship between the allelic variation of *GmCDF1* and salt tolerance. The results showed that only two SNPs, S-671 (located 671 bp upstream of the start codon) and S605 (located 605 bp downstream of the start codon), were significantly associated with ST-GR (Fig 7A), contributing to 20.17% and 32.50% of the phenotypic variations for ST-GR in the representative subset, respectively. The sequencing of *GmCDF1* revealed that S-671 and S605 are located in the promoter region and exon of *GmCDF1*, respectively.

Based on these 11 indels and 15 SNPs, the 31 soybean genotypes were classified into ten haplotypes (Hap1-Hap10) (Fig 7B). Haplotype 1 (Hap1, n = 12) formed the largest group, Hap2 (n = 8) was the second largest group, and the other eight haplotype classes constituted minor groups, each comprising one or two soybean accessions (Fig 7A). The soybeans carrying Hap1 had significantly smaller ST-GR values than those carrying Hap2 (*p* = 8.4×10^−4^) (Fig 7C), which indicated that Hap1 was more sensitive to salt stress than Hap2. With the comparison of these two haplotypes, only two different SNPs, S-671 and S605, which are located in the promoter region and exon of *GmCDF1*, respectively (Fig 7B). However, S605 did not result in an amino acid change. As it is known, the promoter always plays a central role in transcriptional regulation, and the relative expressions of *GmCDF1* were detected in seeds from these 31 soybean accessions treated or not treated with 150 mM NaCl for 48 h. Association mapping was performed using the relative expressions of *GmCDF1*, and 15 polymorphic sites were significantly associated with the relative expressions of *GmCDF1* in these 31 soybean accessions (S7 Table). Except S605, S-671 was the most significant polymorphic sites explaining 21.16% of the phenotypic variation among these polymorphic sites. Moreover, the soybean accessions carrying Hap1 showed higher *GmCDF1* expression than those carrying Hap2 (Fig 7D). These results suggested that S-671, might be the SNP responsible for the difference in relative expressions of *GmCDF1*, leading to different salt tolerance in soybean eventually. Additionally, it was found that the expression of *GmCDF1* was negatively correlated with ST-GR in these 31 soybean accessions (*r* = -0.56, *P* < 0.01). All these results suggested that the expression of *GmCDF1* can partially explain the phenotypic variation in soybean salt tolerance.

## Discussion

Land and water productivities are seriously affected by salt stress, which obviously reduces the food production of major crops, such as rice, wheat and soybean [17,29,30]. Knowledge of the salt tolerance mechanisms in plants is an effective strategy to enhance crop tolerance to salt stress. Whole-genome sequencing was recently shown to be a useful approach for dissecting the genetic architecture of salt tolerance in soybean [15,16]. Here, whole-genome sequencing was employed to refine the QTL for salt tolerance in soybean detected in an analysis combining linkage mapping and GWAS. A series of experiments were then performed to confirm that *GmCDF1* is a negative factor for soybean salt tolerance.

### *GmCDF1* negatively regulates salt tolerance in soybean and is different from its homologs in adaptation to metal stress in plants

Genes encoding members of the CDF family have been cloned from bacteria, yeast, plants, and animals [31–35] and play critical roles in cation accumulation, cation tolerance, signal transduction cascades and oxidative stress resistance [36–39]. In addition, plant CDF transporters usually play an important role in metal homeostasis and tolerance [40]. *AtMTP1* overexpression enhances zinc resistance and accumulation in *Arabidopsis* [41], and *AtMTP3* and *AtMTP11* enhances zinc tolerance and manganese tolerance, respectively [42,43]. In rice, *OsMTP8*.*1* and *OsMTP11* are involved in the uptake and translocation of manganese [38,44]. Two *Beta vulgaris* MTP members, *BmMTP10* and *BmMTP11*, and a cucumber *CsMTP8* have also been identified as manganese transporters that confer increased tolerance to manganese [45,46]. Obviously, these above-mentioned studies of CDF/MTP proteins mainly focused on metal tolerance, such as Zn or Mn tolerance, in plants. In contrast to CDF proteins that are known to improve resistance to metal stress in plants, *GmCDF1* overexpression led to sensitivity to salt stress, whereas the silencing of *GmCDF1* enhanced tolerance to salt stress in soybean. As shown in the present study, *GmCDF1* negatively contributes to the salt adaptation of soybean. To the best of our knowledge, this study provides the first demonstration that *GmCDF1* is negatively associated with salt tolerance in soybean. Notably, the results showed a slight increase in the Zn^2+^ concentration of the *GmCDF1-*OE transgenic soybean hairy roots and a significant reduction in the Zn^2+^ concentration of *GmCDF1-*RNAi transgenic soybean hairy roots compared with their control roots under normal conditions (S9A and S9D Fig), indicating that *GmCDF1* might be involved in Zn^2+^ transport in soybean. In addition, Mn is not involved in *GmCDF1*-regulated salt tolerance because no significant differences in the Mn concentration were found between the transgenic soybean hairy root and non-transgenic soybean hairy roots under either normal or salt stress conditions (S9B and S9E Fig).

### *GmCDF1* regulates salt tolerance through ion homeostasis

The maintenance of ion homeostasis is an important salt tolerance mechanism in soybean. In fact, maintaining an adequate K^+^ concentration and a high K^+^/Na^+^ ratio has been shown to be necessary for plant survival and growth under salt stress [47]. The salt overly sensitive (SOS) signaling pathway has been well characterized for salt tolerance, and *AtSOS1* is indispensable for driving Na^+^ efflux from xylem parenchyma cells to root xylem under salt stress to maintain a relatively low Na^+^ concentration [48]. The overexpression of *GmSOS1* in *A*. *thaliana* reduces Na^+^ accumulation in both the roots and shoots and enhanced tolerance to salt stress at the seed germination and seedling stages [49]. In addition, it has been reported that *A*. *thaliana* Na^+^/H^+^ exchanger 1 (*AtNHX1*) is a Na^+^, K^+^/H^+^ antiporter in *Arabidopsis* [50,51]. In tomato, the overexpression of *AtNHX1* induces the accumulation of K^+^ in vacuoles as well as the transport of K^+^ from roots to shoots [52,53]. Moreover, the overexpression of *GmNHX*1 in *Lotus corniculatus* results in lower Na^+^ and K^+^ contents, a higher K^+^/Na^+^ ratio, and a higher salt tolerance compared with those of wild-type plants under salt stress [54]. The above-mentioned genes are all positively associated with salt tolerance in plants. However, we found a novel gene, *GmCDF1*, that negatively regulated salt tolerance through Na^+^-K^+^ homeostasis in soybean. The overexpression of *GmCDF1* enhanced Na^+^ absorption and depressed the accumulation of K^+^ under salt stress, which led to a higher Na^+^ content and lower K^+^ content in soybean hairy roots compared with those found in the Control 1 roots (Fig 4A and 4B), and the opposite results were obtained with the silencing of *GmCDF1* in soybean hairy roots (Fig 4C and 4D). These results suggest that *GmCDF1* might facilitate the accumulation of Na^+^ and depress the absorption of K^+^, ultimately increasing the ionic toxicity caused by salt stress.

### Crosstalk between *GmCDF1* and two salt tolerance-related genes, *GmSOS1* and *GmNHX1*

Soybean salt tolerance is a complex quantitative trait affected by numerous genetic and non-genetic factors. *GmSOS1* and *GmNHX1* indirectly contribute to soybean tolerance to salt stress [49,54]. Moreover, the silencing of *SlSOS1* in tomato (*Solanum lycopersicum*) results in hypersensitivity to salt stress [55], and the *nhx1* mutation reduces the establishment of *A*. *thaliana* seedlings compared with the wild-type protein under salt stress, indicating that salt tolerance is depressed if *SOS1* or *NHX1* expression is reduced in plants. However, a qRT-PCR analysis of for ion homeostasis-associated genes in transgenic hairy roots showed a negative correlation between the expression of *GmCDF1* and two genes, *GmSOS1* and *GmNHX1*. *GmSOS1* and *GmNHX1* exhibited lower expression in the Control 1 roots than these in the *GmCDF1*-OE hairy roots under salt stress, respectively (Fig 6A and 6B). In contrast, the expression levels of *GmSOS1* and *GmNHX1* were 1.8-fold and 2.0-fold higher, respectively, in the *GmCDF1*-RNAi hairy roots than in the Control 2 hairy roots under salt stress (Fig 6C and 6D). These results suggest that the existence of crosstalk between *GmCDF1* and two salt tolerance-related genes, *GmSOS1* and *GmNHX*1, and the gene expression data indicate that *GmCDF1* negatively regulates salt tolerance in soybean. In addition to *GmSOS1* and *GmNHX1*, other salt tolerance-related genes, such as *GmSALT3*, *GmHKT1;4* and *GmNcl*, were detected in soybean hairy roots under normal and salt conditions, and no significant differences were detected in the expression of these three genes in the *GmCDF1*-OE hairy roots or in the *GmCDF1*-RNAi hairy roots when exposed to salt stress (S10 Fig). Thus, these three genes might not be affected by *GmCDF1*.

In addition, published experimental evidence proves the importance of Ca^2+^ for salt adaptation [56,57]. In this study, the Ca^2+^ concentration in transgenic soybean hairy roots and non-transgenic shoots was not significantly changed compared with that of the control plants under either normal or salt stress conditions (S9C and S9D Fig), indicating that *GmCDF1* might not affect the transportation of Ca^2+^ in soybean. However, the mechanism through which *GmCDF1* regulates the Na^+^-K^+^ balance directly remains to be elucidated in future studies. In recent years, CRISPR/Cas9 technology has been widely used to introduce targeted mutations for studying gene function in plants [58–60], and this powerful tool will be used to explore this gene in our future study.

## Materials and methods

### Plant materials and salt tolerance evaluation

The linkage mapping population consisting of 184 RILs (designated as NJRIKY) was derived from a cross between Kefeng No.1 and Nannong 1138–2 and was developed by single-seed descent at the National Center for Soybean Improvement of China [61,62]. A natural population including 211 cultivated soybean accessions was used for the GWAS (S1 Table). Seeds of the RILs population were collected from four environments: the Jiangpu Experimental Station of the Nanjing Agricultural University (32.12° N 118.37° E), Nanjing, China, in 2012 (E1), 2013 (E2) and 2014 (E3) and the Experimental Farm of the Jiangsu Yanjiang Institute of Agricultural Sciences (31.58° N 120.53° E), Nantong, China, in 2015 (E4). The seeds used for the GWAS were obtained from the following three environments: the Jiangpu Experimental Station of Nanjing Agricultural University (32.12°N 118.37°E) in Nanjing, China, in 2012 and 2013 (E1 and E2, respectively) and the Experimental Farm of the Jiangsu Yanjiang Institute of Agricultural Sciences (31.58°N 120.53°E) in Nantong, China, in 2015 (E4).

Prior to germination, the seeds were sterilized with a chlorine gas atmosphere to minimize the danger of microbial contamination and infection. Forty uniform healthy weighed seeds were then placed on two sheets of filter paper (in sterilized Petri dishes) and treated with 15 mL of water or 150 mM NaCl. The seeds were incubated in a growth chamber at 25±1°C in the dark for 6 days. Twenty-four hours later, the imbibed seeds were weighed to calculate the seed IR. Subsequently, the seeds were placed into new dishes with filter paper, and 5 mL of NaCl solution (0 or 150 mM) was added. After the seeds were rinsed, the number of germinated seeds was counted to calculate the GI, germination potential and GR every day for the next 5 days. Soybean seeds were considered to be germinated when the radicle and plumule length of the soybean seed were greater than the seed length. Three replications were conducted in this study.

The evaluated germination traits included IR [IR (%) = (*W*_2_–*W*_1_)/*W*_1_×100%, where *W*_1_ represents the dry seed weight before imbibition and *W*_2_ represents the seed weight after imbibition for 24 h], GI [GI = Σ(*G*_t_/*D*_t_), where *G*_t_ is the accumulated number of germinated seeds on day t and *D*_t_ indicates the time corresponding to *G*_t_ in days], GP [GP(%) = *N*_*3*_/N*100, where *N*_*3*_ indicates the number of germinated seeds on day 3 and *N* represents the total number of experimental seeds], and GR [GR (%) = *N*_*t*_/N*100%, where *N*_*t*_ indicates the number of germinated seeds on day t and *N* represents the total number of experimental seeds]. The ST was defined as the ratio of the germination-related traits (IR, GI, GR and GP) under salt conditions to the same traits under salt-free conditions [21].

### Phenotypic data analysis

The mean values of all phenotypic data obtained for the RILs population in the four environments and for the natural population in the three environments were utilized for descriptive statistics and correlation analysis. ANOVAs for all traits were performed using SAS 9.0 software (SAS Institute 1999), and Pearson’s correlations between traits were assessed using SPSS 20 software (SPSS Statistics 20). The frequency histograms of the four salt tolerance indices were generated with Origin 8.0 software.

### QTL mapping for salt tolerance

A genetic linkage map was constructed from the 184 F_7:11_ lines of RILs using 221 SSR markers, three EST-SSRs and one *R* gene (resistance to soybean mosaic virus) [61]. The constructed linkage map covered 2,625.9 cM of the soybean genome with an average distance of 11.8 cM between markers. Composite interval mapping (CIM) was employed for QTL mapping with WinQTLCart version 2.5_011 (http://statgen.ncsu.edu/qtlcart/). The control marker number and window size were set to 5 and 10 cM, respectively. The forward and backward regression method was selected, and empirical thresholds were computed using permutation test analyses (1000 permutations, overall error level of 5%) [63]. The QTLs considered to be significant were those with LOD peaks that exceeded the genome-wide threshold of 2.5 [64]. Confidence intervals were defined as the map interval corresponding to a 1-LOD decline on either side of the LOD peak.

### GWAS for salt tolerance

We employed 207,608 SNPs with MAF>5% acquired from the NJAU 355 K SoySNP array to genotype the 211 soybean accessions used in the GWAS performed in this study [25]. The LD decay rate, defined as the chromosomal distance where the LD decays to half of its maximum value, was 130 kb in the 211 cultivated soybeans [25].

The mean values of all phenotypic data from E1, E2, and E3 were used for the GWAS. The GWAS was conducted with an R package called Genome Association and Prediction Integrated Tool (GAPIT) [65] using a compressed mixed linear model (CMLM) and controlling for relatedness and population structure [66]. The threshold for a significant association was set to 1/n (n is the number of markers, *P*≤4.82×10−6 or -log_10_(*P*)≥5.32) [67]. In addition, SNPs within the threshold of 4.5≤-log_10_(*P*)<5.32 were defined as suggestive SNPs [64].

### Whole-genome sequencing of two parents and filtering of candidate genes

Whole-genome sequencing was performed on the two parents of the 184 RILs population, Kefeng No.1 and Nannong 1138–2, using the Illumina HiSeq 4000 sequencing platform. The genome of the cultivated soybean Williams 82 was used as a reference sequence. Genome Analysis Toolkit (GATK) was used for SNP calling to genotype these two soybean accessions.

After obtaining nucleotide polymorphism information from Kefeng No.1 and Nannong 1138–2, we screened the SNPs to obtain candidate genes for salt tolerance. A gene model was considered a candidate gene for salt tolerance if the gene satisfied the following conditions: (1) the SNP was located within a QTL that was found to be significantly associated with salt tolerance in this study and (2) the SNP was located within the coding region of the gene model and resulted in an amino acid exchange or was located in the promoter region of the gene model.

### *Agrobacterium rhizogenes*-mediated transformation of soybean hairy roots

The coding sequence of *GmCDF1* was cloned from NJAU_204, which is tolerant to salt stress, and subsequently subcloned into the vector pMDC83 (containing double CaMV 35S promoter) to produce the pMDC83-*GmCDF1* overexpression vector. Specific primers were employed to amplify a 365-bp fragment from cDNA of NJAU_204 to be ligated into the vector pB7GWIWG2(II), and this ligation yielded the pBI-*GmCDF1* RNAi vector. The primers used to construct these vectors are listed in S4 Table.

pMDC83-*GmCDF1*, pBI-*GmCDF1*-RNAi and their respective empty vectors were independently transformed into *Agrobacterium rhizogenes* strain K599 for hairy root transformation [28]. One-week-old seedlings were injected with transformed K599 and transferred to a temperature-controlled germination chamber with a 12-h light/12-h dark cycle, a day/night temperature of 28°C/25°C and high humidity. Approximately two to three weeks later, specifically when the hairy roots were approximately 2–10 cm near the infection site where the hairy roots formed, the primary root was cut off. The hairy roots of the seedlings were immersed in 1/2 Hoagland nutrient solution for five days and then treated with water or 75 mM NaCl for four days. In addition, the chlorophyll concentrations of the top secondary fully expanded leaves were measured three times using a chlorophyll meter (Konica Minolta SPAD502) and are expressed as SPAD values, and the fresh weights of the hairy roots from all the transgenic line were noted prior to PCR confirmation. Subsequently, the soybean hairy roots and shoots were harvested separately and used for gene expression or ICP-OES analysis. Negative soybean hairy roots and their respective shoots were discarded as soon as their phenotypic data were collected, and the samples for ICP-OES analysis were rinsed three times with deionized water.

### Quantitative RT-PCR for determining the expression level of genes

To analyze the expression of candidate genes, seeds of 31 soybean accessions including the two parents (Kefeng No.1 and Nannong 1138–2) of the RILs population were sterilized with a chlorine gas atmosphere to minimize the danger of microbial contamination and infection and treated with 0 or 150 mM NaCl as in the above-described seed germination experiment. After treatment with or without salt stress for 48 h, 15 quarters of the embryos from three replications were sampled and stored at -80°C for the isolation of total RNA.

To analyze the expression of *GmCDF1* in different soybean tissues, we sampled different tissues from the roots, stems, leaves, and flowers during the full-blossom period, pod walls on the 15th day after flowering (DAF), and seeds at 15 DAF.

Total RNA was isolated using the RNAsimple Total RNA Kit (TIANGEN Beijing, China), and first-strand cDNA was reverse-transcribed using a TaKaRa Primer Script RT reagent kit with gDNA Eraser. Gene expression was determined by RT-PCR using an ABI 7500 system (Applied Biosystems, Foster City, CA, USA) with the SYBR Green Real-time Master Mix (Toyobo), and the data were analyzed using ABI 7500 Sequence Detection System (SDS) software version 1.4.0. The normalized expression was calculated for each sample as ΔΔC_T_ = (C_T, Target_-C_T, Tubulin_)_genotype_-(C_T, Target_-C_T, Tubulin_)_calibrator_, and the fold change was calculated as 2^-ΔΔCT^ [68]. The transcript level of *tubulin* (GenBank accession number: AY907703) was used as a quantitative control. The primers used in the present study are listed in S2 Table.

### ICP-OES analysis

Samples of hairy roots and shoots were dried at 105°C for 60 min and then dried at 65°C for 72 h in a forage dryer. Then, 50–100 mg of each dry sample was weighed, and 2 mL of HNO_3_ and 8 mL of deionized water were added. The mixtures were digested at 200°C for 10 min using an Ethos Microwave Digestion Labstation (Milestone Rrl., Sorisole, Italy). After cooling, the digested samples were diluted to 50 mL with distilled water. The contents of Na^+^, Ca^2+^, K^+^ Zn^2+^ and Mn^2+^ were then detected using an Optima 8000 DV Inductively Coupled Plasma Optical Emission Spectrometer (ICP-OES) system (PerkinElmer Inc., Waltham, MA, USA).

### *GmCDF1*-based association analysis

The genome sequence of *GmCDF1* from 31 soybean genotypes (S5 Table) was amplified using the specific primers described in S2 Table. The sequences were assembled and aligned using ContigExpress in Vector NTI Advance 10 (Invitrogen, Carlsbad, CA, USA) and MEGA version 6 [69], respectively. Polymorphisms with MAF>0.05, including SNPs and indels, were identified among these genotypes, and their association with salt tolerance indices was calculated with Tassel 5.0 [70]. The analysis of *GmCDF1* haplotypes and the pairwise LD analysis were performed with Haploview 4.2 [71]. Markers were defined as being significantly associated with the phenotype based on the significant association thresholds of -log_10_(*P*)*>*1.30 and *P<*0.05.

## Supporting information

S1 Fig **Frequency distributions of four salt tolerance indices (ST-IR, ST-GI, ST-GP and ST-GR) in recombinant inbred lines (RILs) (left column) and natural populations (right column) based on the means of the traits obtained in four and three environments, respectively**.
**(A)** and **(E)** ST-IR: ratio of the imbibition rate under salt conditions to the imbibition rate under no-salt conditions;
**(B)** and **(F)** ST-GI: ratio of the germination index under salt conditions to the germination index under no-salt conditions;
**(C)** and **(G)** ST-GP: ratio of the germination potential under salt conditions to the germination index under no-salt conditions;
**(D)** and **(H)** ST-GR: ratio of the germination rate under salt conditions to the germination rate under no-salt conditions.
(TIF)

S2 Fig SNP density within the soybean genome. The SNP distribution is mapped to the reference genome Williams 82. Outermost circle: 20 chromosomes of soybean; second circle: indel distribution; third circle: distribution of SNPs between the parental lines (Kefeng No.1 and Nannong 1138–2); fourth circle: distribution of GC-skew within the soybean genome; innermost circle: distribution of GC content.
(TIF)

S3 Fig Quantile-quantile plots of the GWAS results for the ST-IR, ST-GI, ST-GP, and ST-GR in three environments (E1, E2, and E3). The three rows from the top to the bottom show the quantile-quantile plots of the GWAS results for the four salt tolerance indices in E1, E2 and E3, respectively.
(TIF)

S4 Fig Relative expression of 16 genes in the parents of the RILs population, Kefeng No.1 (tolerant) and Nannong 1138–2 (sensitive), after salt treatment for 48 h. The Y-axis denotes the gene expression levels. The qRT-PCR results were normalized with to the *tubulin* reference gene. The error bars indicate the SEs of three replicates. Statistical significance was detected by a two-tailed t-test.
(TIF)

S5 Fig Relative expression of seven genes in the parents of the RILs population, Kefeng No.1 (tolerant) and Nannong 1138–2 (sensitive), after salt treatment for 48 h. (gene annotations: *Glyma*.*102000*, cation efflux family protein; *Glyma*.*102800*, protein of unknown function; *Glyma*.*104500*, protein of unknown function; *Glyma*.*08g105200*, calmodulin-binding transcription activator 4-like; *Glyma*.*08g105800*, MAC/perforin domain-containing protein; *Glyma*.*08g106000*, amidase family protein; and *Glyma*.*08g106000*, ribosomal protein S19). The Y-axis denotes the gene expression level. The qRT-PCR results were normalized to the *tubulin* reference gene. The error bars show the SEs of three replicates. Statistical significance was detected by a two-tailed t-test.
(TIF)

S6 Fig Bioinformatics analyses of *GmCDF1* nucleotide and amino acid sequences. **(A)** Phylogenetic tree of the MTP family from rice and *Arabidopsis* and *GmCDF1*. The tree was constructed using MEGA 6.0 with the neighbor-joining method. The *Arabidopsis* MTP amino acid sequences were obtained from (www.tigr.org): *AtMTP1*, *At2g46800*; *AtMTP2*, *At3g61940*; *AtMTP3*, *At3g58810*; *AtMTP4*, *At2g29410*; *AtMTP5*, *At3g12100*; *AtMTP6*, *At2g47830*; *AtMTP7*, *At1g51610*; *AtMTP8*, *At3g58060*; *AtMTP9*, *At1g79520*; *AtMTP10*, *At1g16310*; *AtMTP11*, *At2g39450*; *AtMTP12*, *At2g04620*.
The rice MTP amino acid sequences were downloaded from (http://rice.plantbiology.msu.edu/): *OsMTP1*, *Os05g03780*; *OsMTP5*, *Os02g58580*; *OsMTP6*, *Os03g22550*; *OsMTP7*, *Os04g23180*; *OsMTP8*, *Os02g53490*; *OsMTP8*.*1*, *Os03g12580*; *OsMTP9*, *Os01g03914*; *OsMTP11*, *Os01g62070*; *OsMTP11*.*1*, *Os05g38670*; *OsMTP12*, *Os08g32680*.
The amino acid sequence of Gm*CDF1* (*Glma*.*08g102000*) was downloaded from phytozome (https://phytozome.jgi.doe.gov/pz/portal.html).
**(B)** Amino acid alignment of *GmCDF1* and *AtMTP12*. The amino acid sequences of 14 predicted transmembrane (TM) segments are underlined. The amino acid residues with red shading indicate those conserved in two protein sequences.
(TIF)

S7 Fig Relative expression of *GmCDF1* in different soybean tissues. The bars represent the standard errors from three technical replicates of three biological replicates. The qRT-PCR results were normalized to the *tubulin* reference gene.
(TIF)

S8 Fig Relative expression of *GmCDF1* in soybean hairy roots. **(A)** Relative expression of *GmCDF1* in *GmCDF1*–OE hairy roots and Control 1 roots after treatment with 0 mM or 75 mM NaCl for four days. **(B)** *GmCDF1*-RNAi hairy roots and Control 2 roots. The data are presented as the means±SEs (n≥3). The qRT-PCR results were normalized to the *tubulin* reference gene. Each experiment was performed more than three times with similar results.
(TIF)

S9 Fig Contents of Zn^2+^, Ca^2+^ and Mn^2+^ in soybean hairy roots and shoots under salt stress. **(A)** Zn^2+^ contents of *GmCDF1*-OE hairy roots and their nontransgenic shoots after treatment with 0 mM or 75 mM NaCl for 4 days compared with those of Control 1 plants.
**(B)** Zn^2+^ contents of *GmCDF1*-RNAi hairy roots and their nontransgenic shoots after treatment with 0 mM or 75 mM NaCl for 4 days compared with those of Control 2 plants.
**(C)** Ca^2+^ contents of *GmCDF1*-OE hairy roots and their nontransgenic shoots after treatment with 0 mM or 75 mM NaCl for 4 days compared with those of Control 1 plants.
**(D)** Ca^2+^ contents of *GmCDF1*-RNAi hairy roots and their nontransgenic shoots after treatment with 0 mM or 75 mM NaCl for 4 days compared with those of Control 2 plants.
(E) Mn^2+^ contents of *GmCDF1*-OE hairy roots and their nontransgenic shoots after treatment with 0 mM or 75 mM NaCl for 4 days compared with those of Control 1 plants.
(F) Mn^2+^ contents of *GmCDF1*-RNAi hairy roots and their nontransgenic shoots after treatment with 0 mM or 75 mM NaCl for 4 days compared with those of Control 2 plants.
(* p<0.05, **p<0.01)
(TIF)

S10 Fig Relative expression levels of *GmSALT3*, *GmHKT1;4* and *GmNcl* in soybean hairy roots. No significant differences were detected in the expression of these three genes in the *GmCDF1*-OE hairy roots or in the *GmCDF1*-RNAi hairy roots when exposed to salt stress. (**A**) Relative expression levels of *GmSALT3* in Control 1 and *GmCDF1*-OE hairy roots after treatment with normal or salt conditions for four days. The data are presented as the means±SEs (n≥3). Statistical significance was detected by a two-tailed t-test. (**B**) Relative expression levels of *GmHKT1;4* in Control 1 and *GmCDF1*-OE hairy roots after treatment with normal or salt conditions for four days. The values are the means±SEs (n≥3). Statistical significance was detected by a two-tailed t-test. (**C**) Relative expression levels of *GmNcl* in Control 1 and *GmCDF1*-OE hairy roots after treatment with normal or salt conditions for four days. The data are presented as the means±SEs (n≥3). Statistical significance was detected by a two-tailed t-test. (**D**) Relative expression levels of *GmSALT3* in Control 2 and *GmCDF1*-RNAi hairy roots after treatment with normal or salt conditions for four days. The data are presented as the means±SEs (n≥3). Statistical significance was detected by a two-tailed t-test. (**E**) Relative expression levels of *GmHKT1;4* in Control 2 and *GmCDF1*-RNAi hairy roots after treatment with normal or salt conditions for four days. The data are presented as the means±SEs (n≥3). Statistical significance was detected by a two-tailed t-test. (**F**) Relative expression levels of *GmNcl* in Control 2 and *GmCDF1*-RNAi hairy roots after treatment with normal or salt conditions for four days. The data are presented as the means±SEs (n≥3). Statistical significance was detected by a two-tailed t-test.
(TIF)

S1 Table Descriptive statistics and ANOVA of three germination-related traits under 0 mM NaCl (C) or 150 mM NaCl (S) conditions and four salt tolerance indices based on the means of the traits in the parents, 184 recombinant inbred lines (RILs) and 211 soybean accessions. (XLSX)

S2 Table Phenotypic correlations between the four salt tolerance indices based on the means of the traits in 184 recombinant inbred lines (RILs) and 211 soybean accessions. (XLSX)

S3 Table Details of SNPs significantly associated with four salt tolerance indices of soybean (with the suggestive threshold of 4.5≤-log_10_(*P*)< 5.32). (XLSX)

S4 Table SNPs existing in exons and within the 2.0-kb promoter regions located on chromosome 8 between the SNP markers AX-93912074 and AX-93634504. (XLSX)

S5 Table Single nucleotide polymorphisms (SNPs) of *GmCDF1* in 31 soybean accessions. (XLSX)

S6 Table The relative expression of *GmCDF1* in 31 soybean accessions and the ST-GR of 31 soybean accessions across three environments. (XLSX)

S7 Table Significant SNPs associated with relative expression of GmCDF1 in the 31 soybean accessions (significant association thresholds of -log_10_(*P*)>1.30 and *P*≤0.05). (XLSX)

S8 Table Summary of 211 soybean accessions. (XLSX)

S9 Table Primers used in this study. (XLSX)
