# Supplementary figures and images for: A cation diffusion facilitator, GmCDF1, negatively regulates salt tolerance in soybean
Source: PLoS Genet. 2019 Jan 7;15(1):e1007798. doi: 10.1371/journal.pgen.1007798 (PMC6336350; doi:10.1371/journal.pgen.1007798)

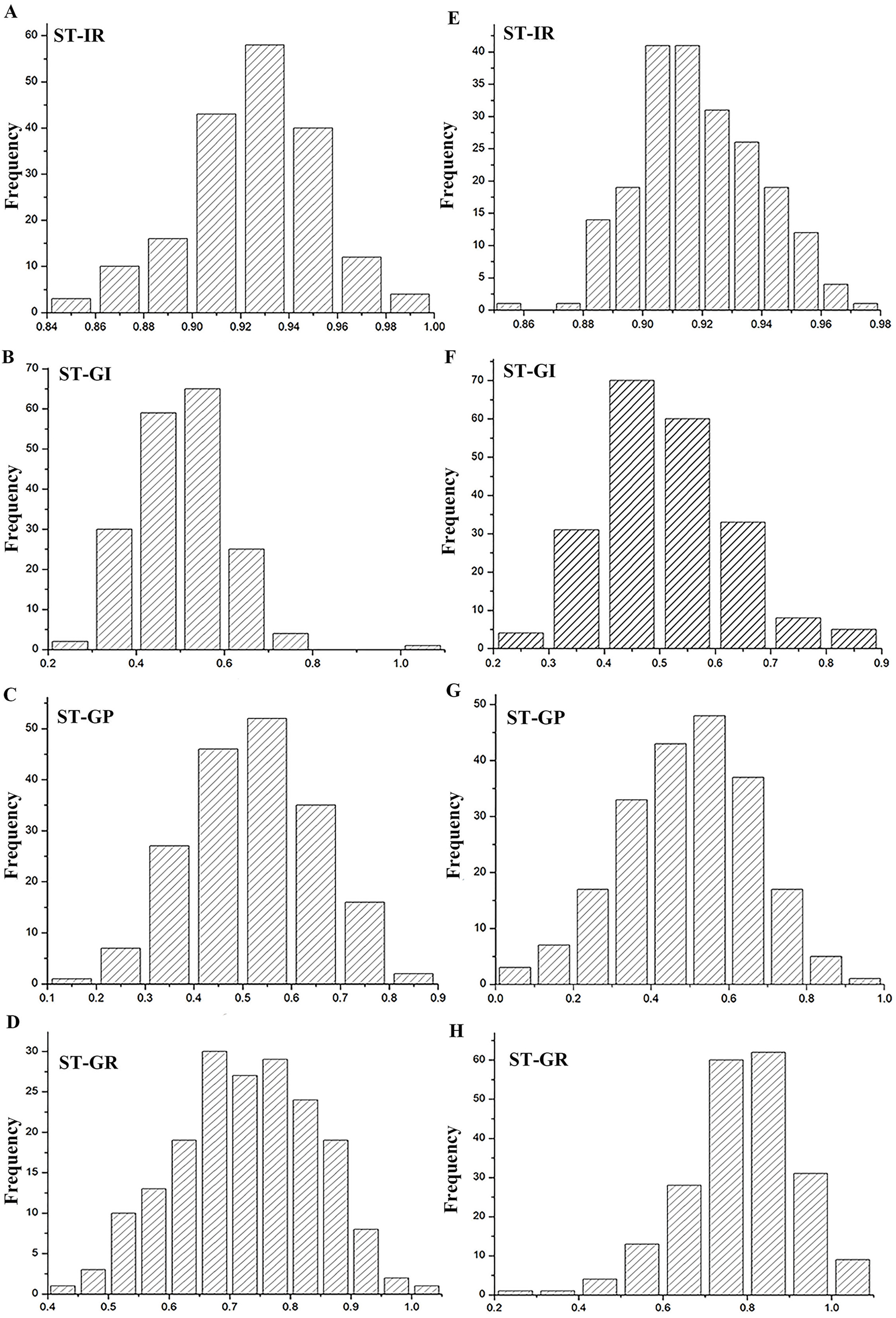

Supplement: S1 Fig — Frequency distributions of four salt tolerance indices (ST-IR, ST-GI, ST-GP and ST-GR) in recombinant inbred lines (RILs) (left column) and natural populations (right column) based on the means of the traits obtained in four and three environments, respectively. (A) and (E) ST-IR: ratio of the imbibition rate under salt conditions to the imbibition rate under no-salt conditions; (B) and (F) ST-GI: ratio of the germination index under salt conditions to the germination index under no-salt conditions; (C) and (G) ST-GP: ratio of the germination potential under salt conditions to the germination index under no-salt conditions; (D) and (H) ST-GR: ratio of the germination rate under salt conditions to the germination rate under no-salt conditions. (TIF) [file pgen.1007798.s001.tif]

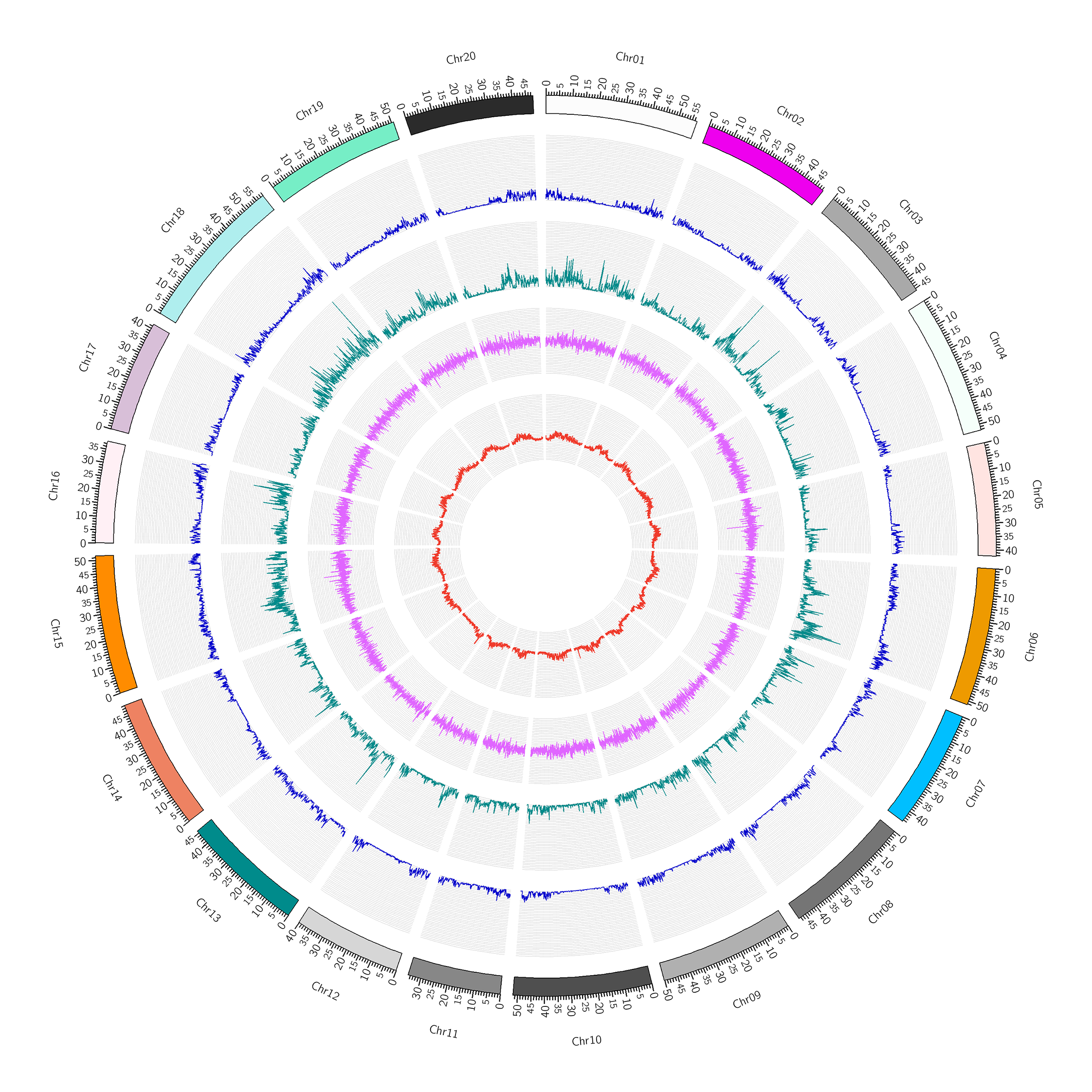

Supplement: S2 Fig — The SNP distribution is mapped to the reference genome Williams 82. Outermost circle: 20 chromosomes of soybean; second circle: indel distribution; third circle: distribution of SNPs between the parental lines (Kefeng No.1 and Nannong 1138–2); fourth circle: distribution of GC-skew within the soybean genome; innermost circle: distribution of GC content. (TIF) [file pgen.1007798.s002.tif]

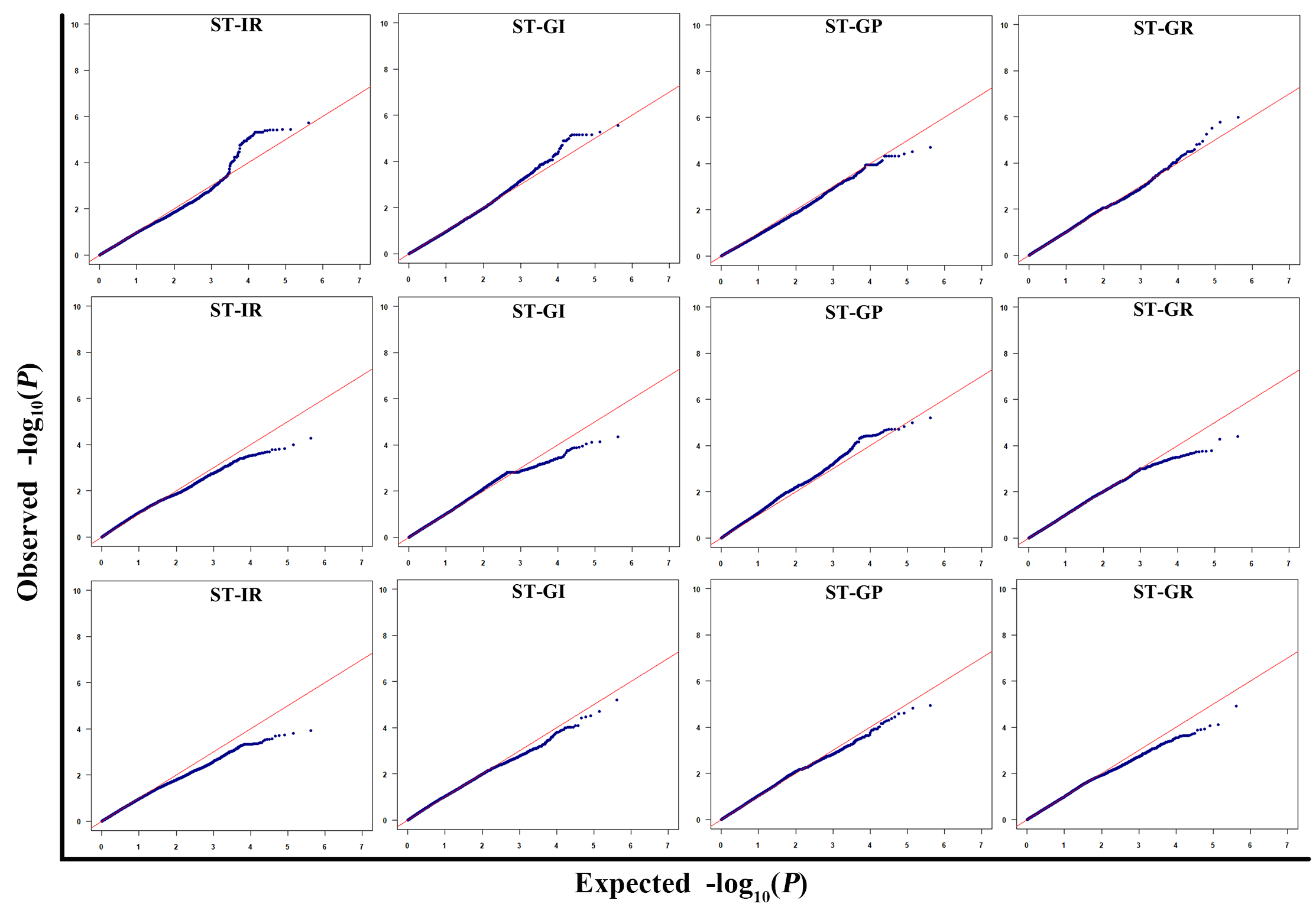

Supplement: S3 Fig — The three rows from the top to the bottom show the quantile-quantile plots of the GWAS results for the four salt tolerance indices in E1, E2 and E3, respectively. (TIF) [file pgen.1007798.s003.tif]

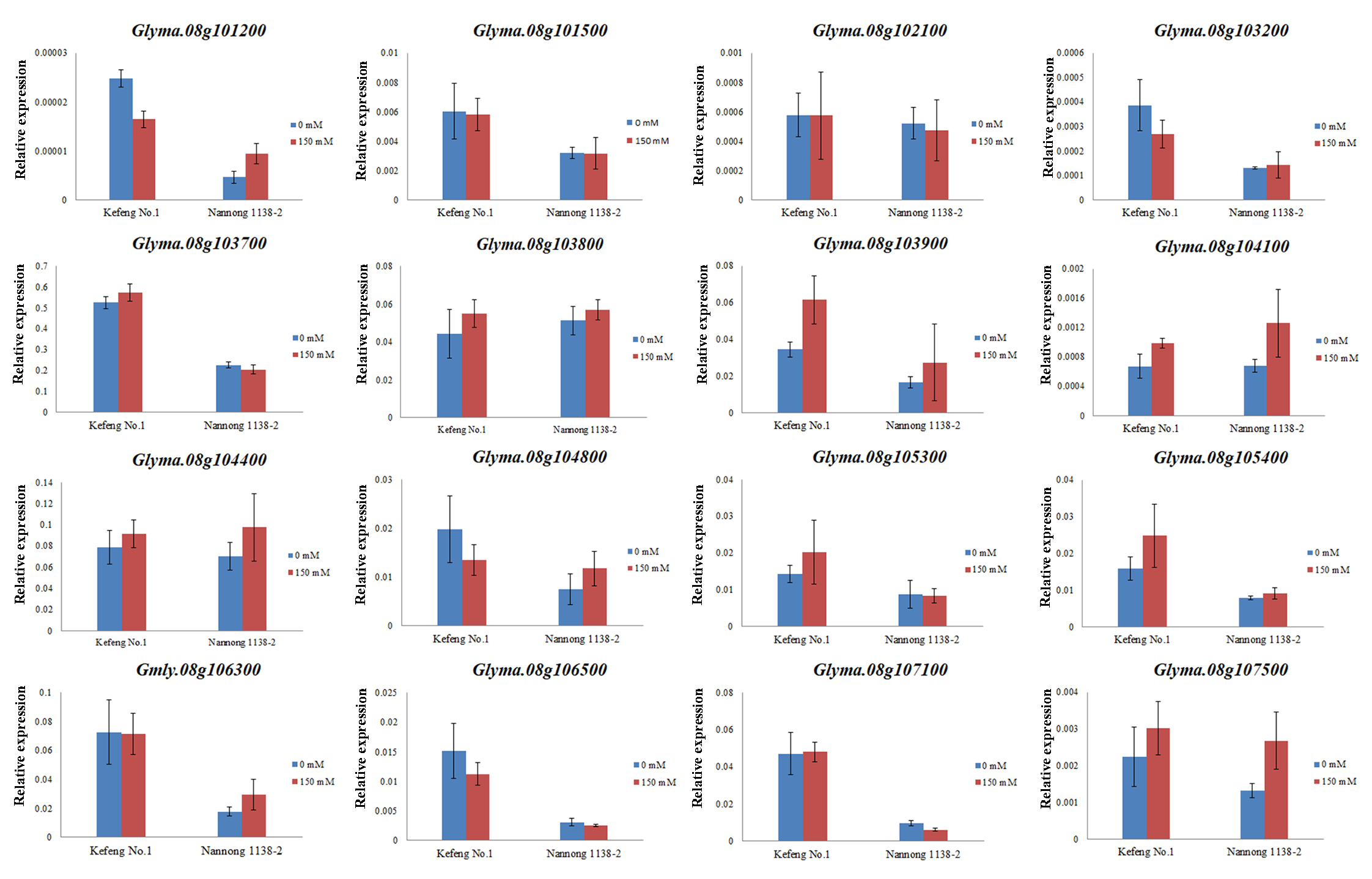

Supplement: S4 Fig — The Y-axis denotes the gene expression levels. The qRT-PCR results were normalized with to the tubulin reference gene. The error bars indicate the SEs of three replicates. Statistical significance was detected by a two-tailed t-test. (TIF) [file pgen.1007798.s004.tif]

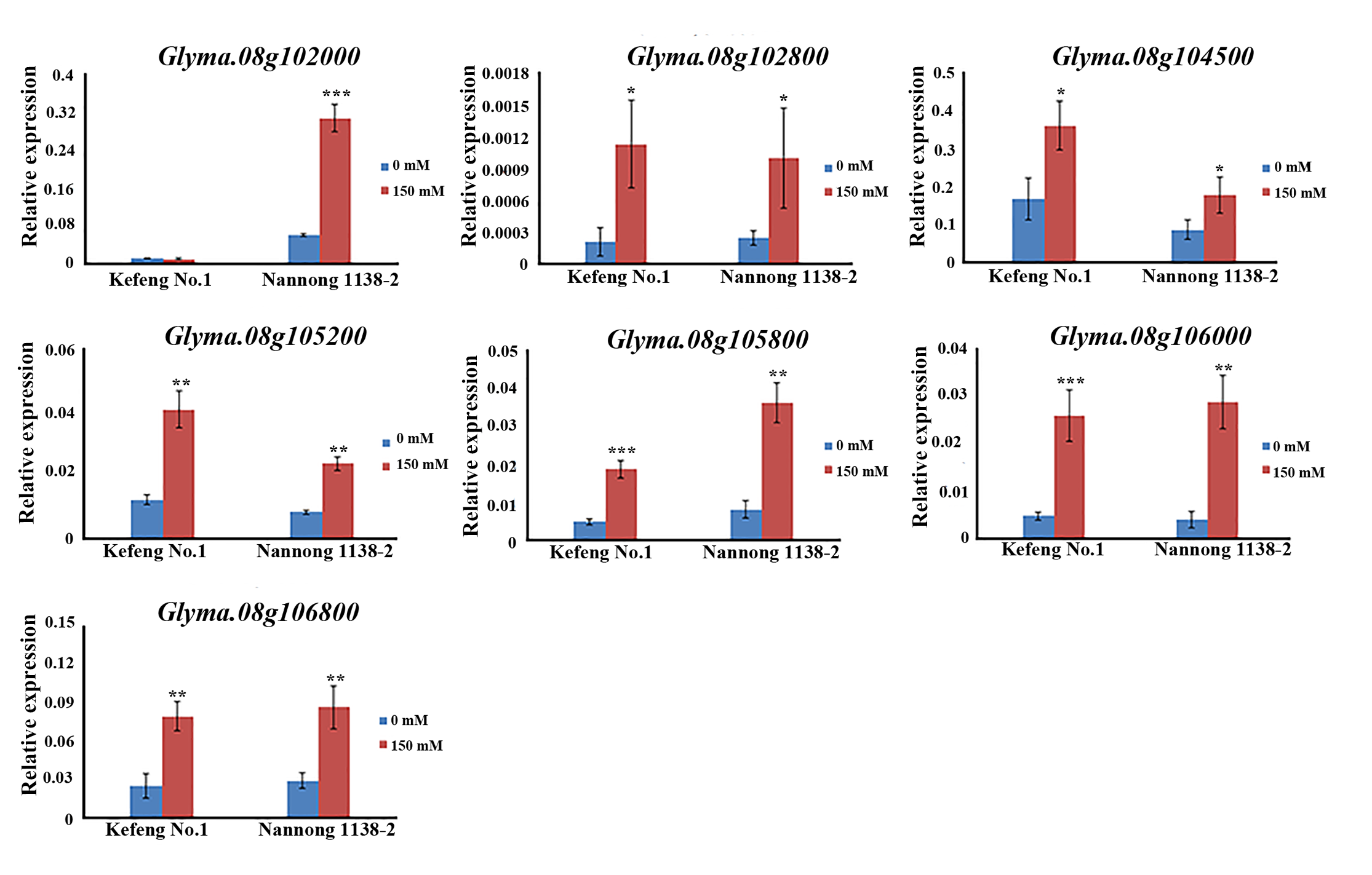

Supplement: S5 Fig — (gene annotations: Glyma.102000, cation efflux family protein; Glyma.102800, protein of unknown function; Glyma.104500, protein of unknown function; Glyma.08g105200, calmodulin-binding transcription activator 4-like; Glyma.08g105800, MAC/perforin domain-containing protein; Glyma.08g106000, amidase family protein; and Glyma.08g106000, ribosomal protein S19). The Y-axis denotes the gene expression level. The qRT-PCR results were normalized to the tubulin reference gene. The error bars show the SEs of three replicates. Statistical significance was detected by a two-tailed t-test. (TIF) [file pgen.1007798.s005.tif]

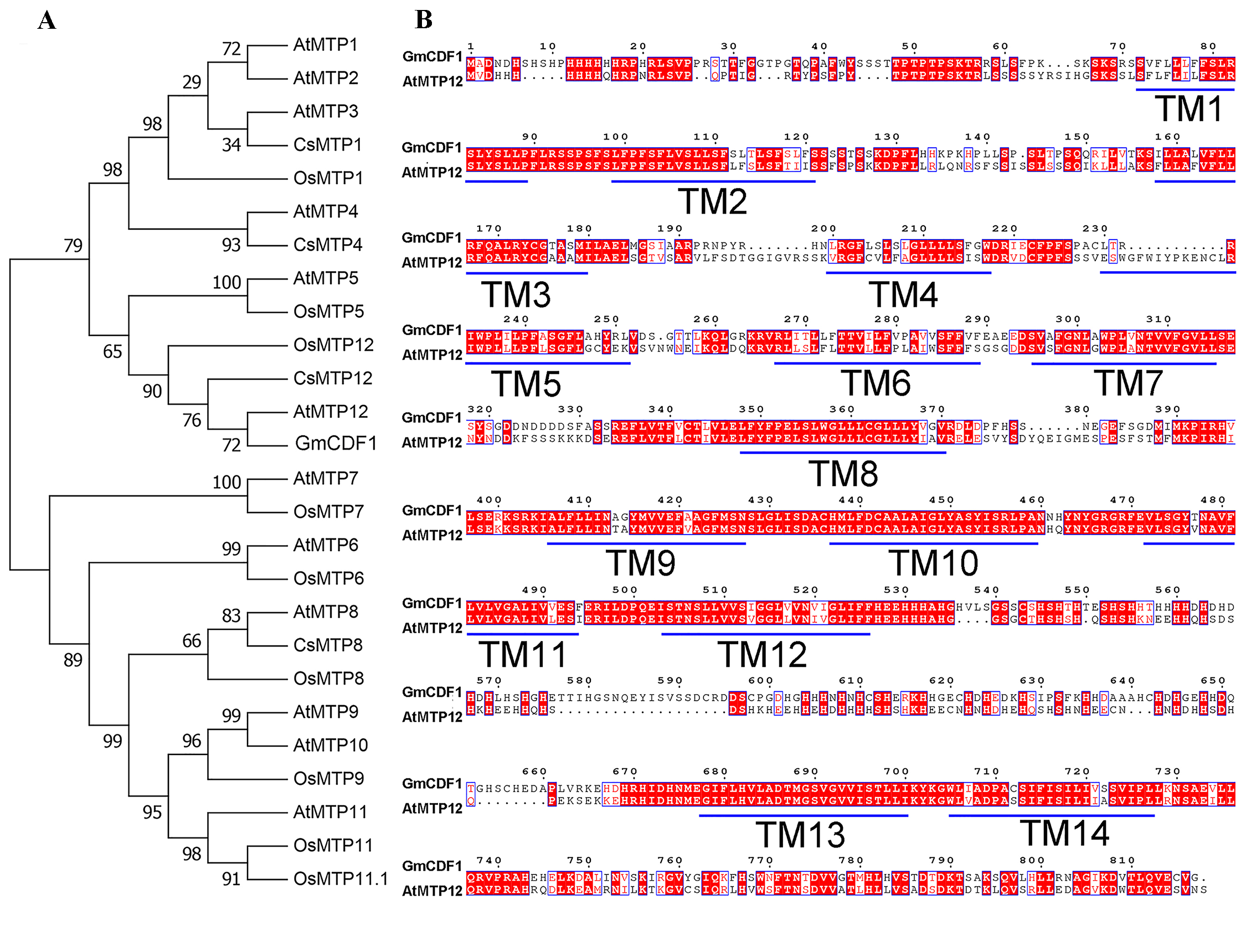

Supplement: S6 Fig — (A) Phylogenetic tree of the MTP family from rice and Arabidopsis and GmCDF1. The tree was constructed using MEGA 6.0 with the neighbor-joining method. The Arabidopsis MTP amino acid sequences were obtained from (www.tigr.org): AtMTP1, At2g46800; AtMTP2, At3g61940; AtMTP3, At3g58810; AtMTP4, At2g29410; AtMTP5, At3g12100; AtMTP6, At2g47830; AtMTP7, At1g51610; AtMTP8, At3g58060; AtMTP9, At1g79520; AtMTP10, At1g16310; AtMTP11, At2g39450; AtMTP12, At2g04620. The rice MTP amino acid sequences were downloaded from (http://rice.plantbiology.msu.edu/): OsMTP1, Os05g03780; OsMTP5, Os02g58580; OsMTP6, Os03g22550; OsMTP7, Os04g23180; OsMTP8, Os02g53490; OsMTP8.1, Os03g12580; OsMTP9, Os01g03914; OsMTP11, Os01g62070; OsMTP11.1, Os05g38670; OsMTP12, Os08g32680. The amino acid sequence of GmCDF1 (Glma.08g102000) was downloaded from phytozome (https://phytozome.jgi.doe.gov/pz/portal.html). (B) Amino acid alignment of GmCDF1 and AtMTP12. The amino acid sequences of 14 predicted transmembrane (TM) segments are underlined. The amino acid residues with red shading indicate those conserved in two protein sequences. (TIF) [file pgen.1007798.s006.tif]

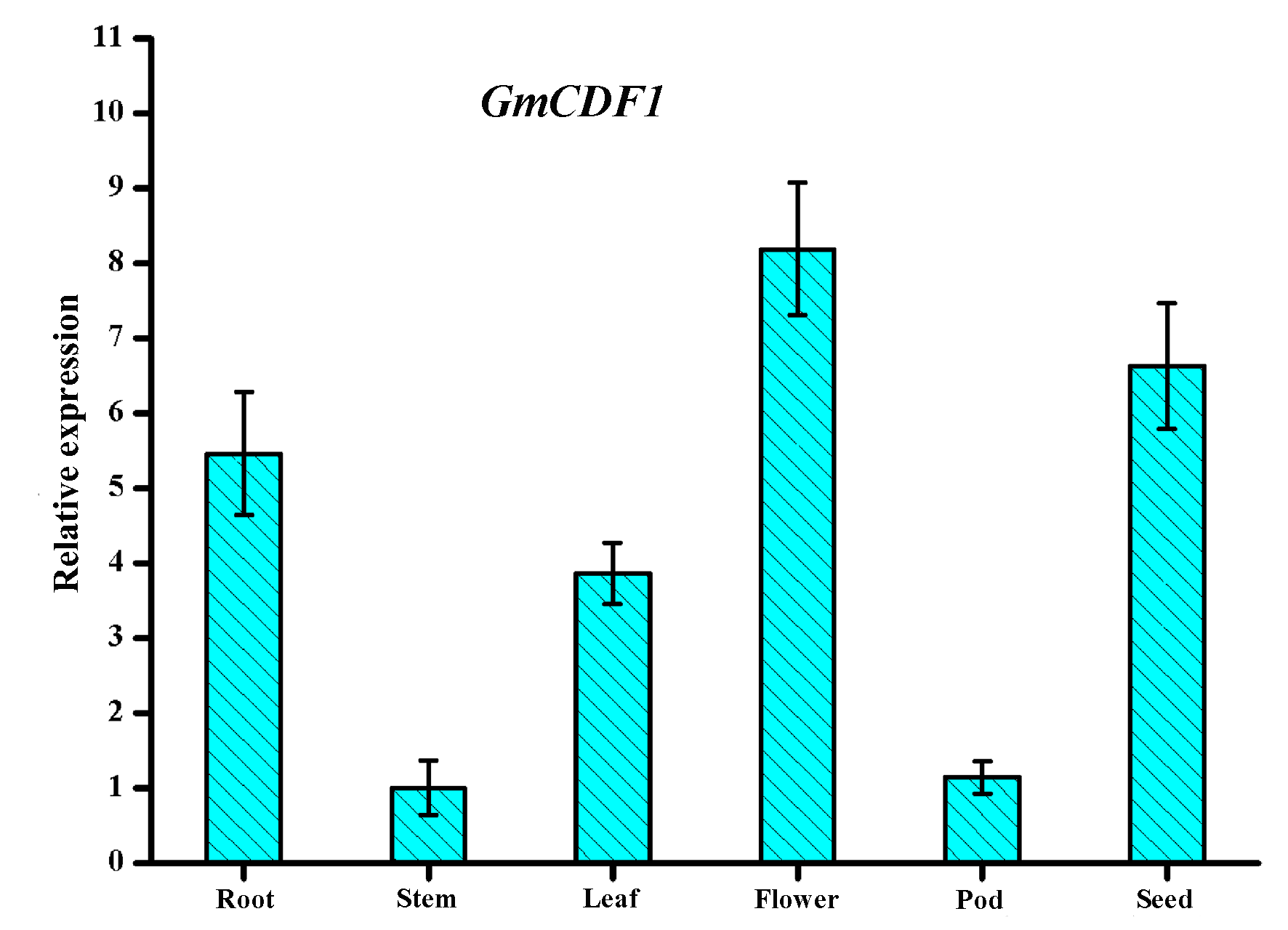

Supplement: S7 Fig — The bars represent the standard errors from three technical replicates of three biological replicates. The qRT-PCR results were normalized to the tubulin reference gene. (TIF) [file pgen.1007798.s007.tif]

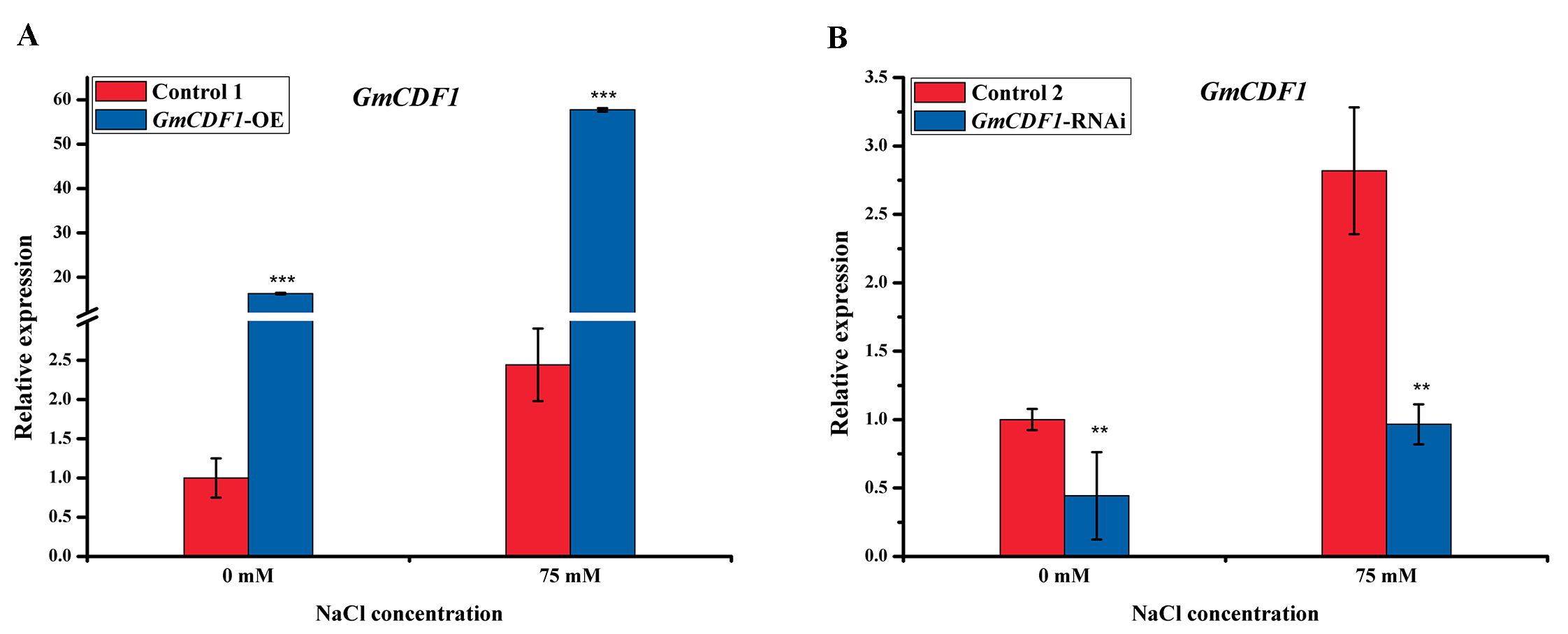

Supplement: S8 Fig — (A) Relative expression of GmCDF1 in GmCDF1–OE hairy roots and Control 1 roots after treatment with 0 mM or 75 mM NaCl for four days. (B) GmCDF1-RNAi hairy roots and Control 2 roots. The data are presented as the means±SEs (n≥3). The qRT-PCR results were normalized to the tubulin reference gene. Each experiment was performed more than three times with similar results. (TIF) [file pgen.1007798.s008.tif]

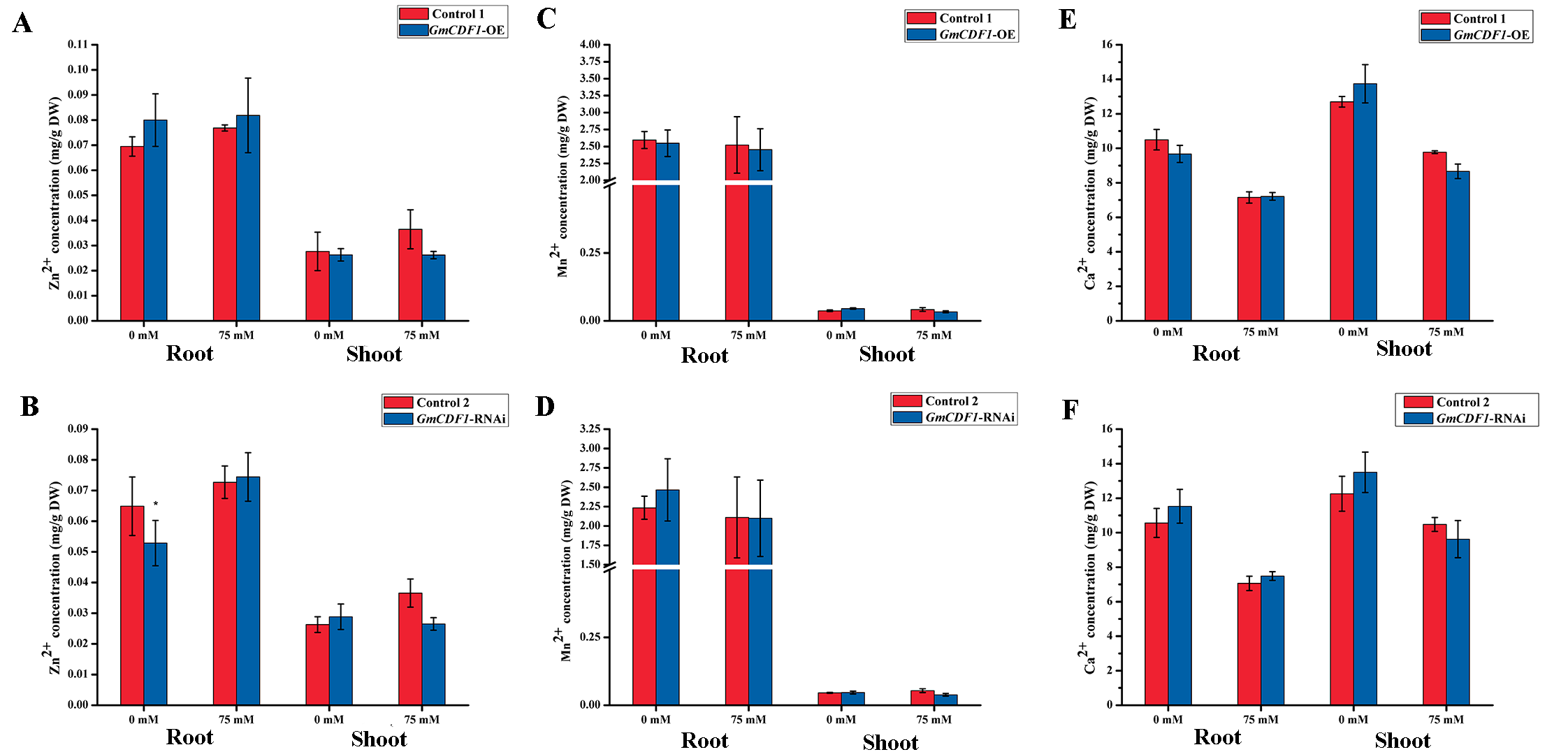

Supplement: S9 Fig — (A) Zn2+ contents of GmCDF1-OE hairy roots and their nontransgenic shoots after treatment with 0 mM or 75 mM NaCl for 4 days compared with those of Control 1 plants. (B) Zn2+ contents of GmCDF1-RNAi hairy roots and their nontransgenic shoots after treatment with 0 mM or 75 mM NaCl for 4 days compared with those of Control 2 plants. (C) Ca2+ contents of GmCDF1-OE hairy roots and their nontransgenic shoots after treatment with 0 mM or 75 mM NaCl for 4 days compared with those of Control 1 plants. (D) Ca2+ contents of GmCDF1-RNAi hairy roots and their nontransgenic shoots after treatment with 0 mM or 75 mM NaCl for 4 days compared with those of Control 2 plants. (E) Mn2+ contents of GmCDF1-OE hairy roots and their nontransgenic shoots after treatment with 0 mM or 75 mM NaCl for 4 days compared with those of Control 1 plants. (F) Mn2+ contents of GmCDF1-RNAi hairy roots and their nontransgenic shoots after treatment with 0 mM or 75 mM NaCl for 4 days compared with those of Control 2 plants. (* p<0.05, **p<0.01) (TIF) [file pgen.1007798.s009.tif]

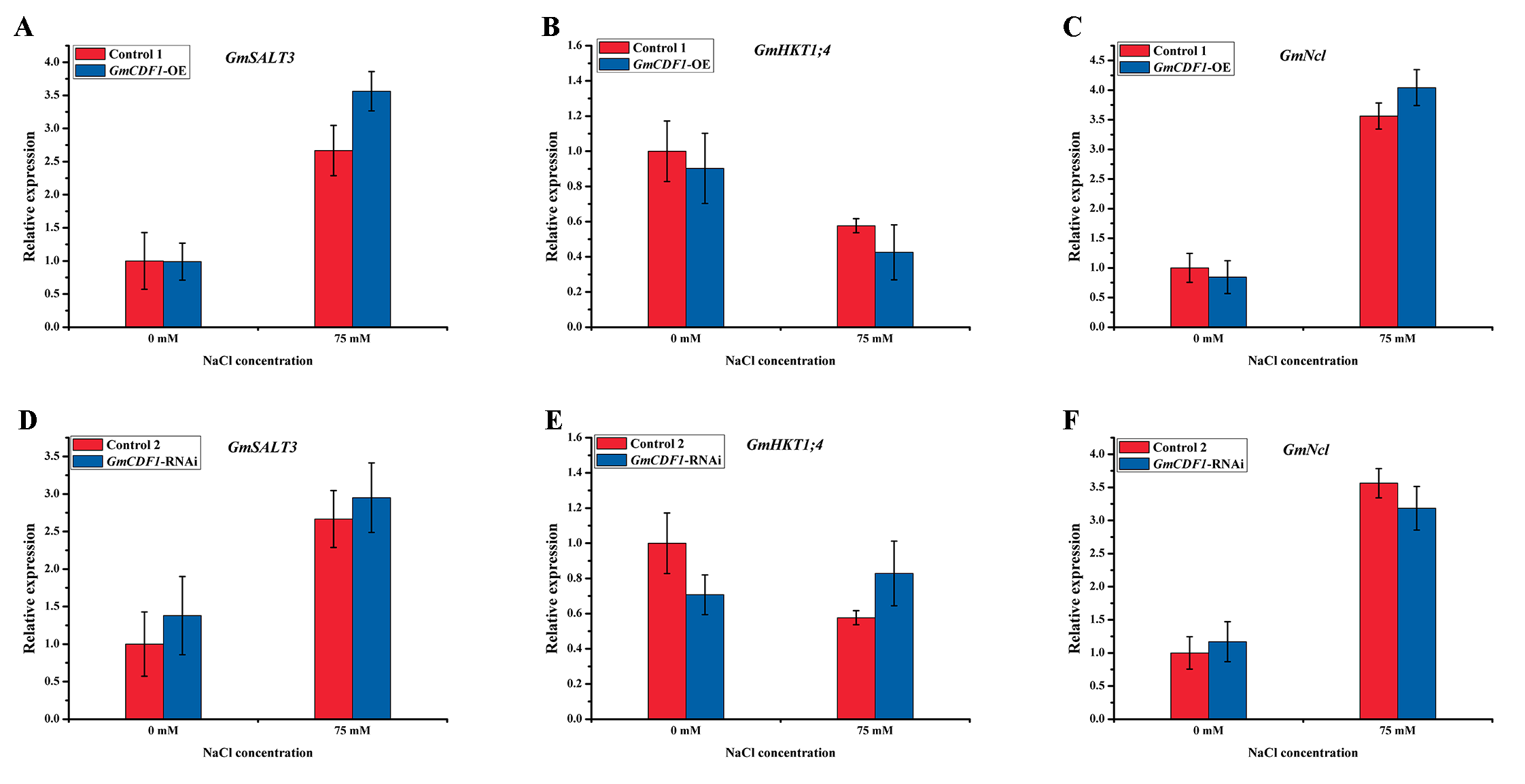

Supplement: S10 Fig — No significant differences were detected in the expression of these three genes in the GmCDF1-OE hairy roots or in the GmCDF1-RNAi hairy roots when exposed to salt stress. (A) Relative expression levels of GmSALT3 in Control 1 and GmCDF1-OE hairy roots after treatment with normal or salt conditions for four days. The data are presented as the means±SEs (n≥3). Statistical significance was detected by a two-tailed t-test. (B) Relative expression levels of GmHKT1;4 in Control 1 and GmCDF1-OE hairy roots after treatment with normal or salt conditions for four days. The values are the means±SEs (n≥3). Statistical significance was detected by a two-tailed t-test. (C) Relative expression levels of GmNcl in Control 1 and GmCDF1-OE hairy roots after treatment with normal or salt conditions for four days. The data are presented as the means±SEs (n≥3). Statistical significance was detected by a two-tailed t-test. (D) Relative expression levels of GmSALT3 in Control 2 and GmCDF1-RNAi hairy roots after treatment with normal or salt conditions for four days. The data are presented as the means±SEs (n≥3). Statistical significance was detected by a two-tailed t-test. (E) Relative expression levels of GmHKT1;4 in Control 2 and GmCDF1-RNAi hairy roots after treatment with normal or salt conditions for four days. The data are presented as the means±SEs (n≥3). Statistical significance was detected by a two-tailed t-test. (F) Relative expression levels of GmNcl in Control 2 and GmCDF1-RNAi hairy roots after treatment with normal or salt conditions for four days. The data are presented as the means±SEs (n≥3). Statistical significance was detected by a two-tailed t-test. (TIF) [file pgen.1007798.s010.tif]
